# Toward trustworthy medical device *in silico* clinical trials: a hierarchical framework for establishing credibility and strategies for overcoming key challenges

**DOI:** 10.3389/fmed.2024.1433372

**Published:** 2024-08-12

**Authors:** Kenneth I. Aycock, Tom Battisti, Ashley Peterson, Jiang Yao, Steven Kreuzer, Claudio Capelli, Sanjay Pant, Pras Pathmanathan, David M. Hoganson, Steve M. Levine, Brent A. Craven

**Affiliations:** ^1^Office of Science and Engineering Laboratories, Center for Devices and Radiological Health, United States Food and Drug Administration, Silver Spring, MD, United States; ^2^Dassault Systèmes, Waltham, MA, United States; ^3^Thornton Tomasetti, Inc., New York, NY, United States; ^4^Exponent, Inc., Menlo Park, CA, United States; ^5^Institute of Cardiovascular Science, University College London, London, United Kingdom; ^6^Faculty of Science and Engineering, Swansea University, Swansea, United Kingdom; ^7^Department of Cardiac Surgery, Boston Children’s Hospital, Harvard Medical School, Boston, MA, United States

**Keywords:** *In silico* clinical trial, ISCT, model credibility, computational modeling and simulation, hierarchical verification and validation

## Abstract

Computational models of patients and medical devices can be combined to perform an *in silico* clinical trial (ISCT) to investigate questions related to device safety and/or effectiveness across the total product life cycle. ISCTs can potentially accelerate product development by more quickly informing device design and testing or they could be used to refine, reduce, or in some cases to completely replace human subjects in a clinical trial. There are numerous potential benefits of ISCTs. An important caveat, however, is that an ISCT is a virtual representation of the real world that has to be shown to be credible before being relied upon to make decisions that have the potential to cause patient harm. There are many challenges to establishing ISCT credibility. ISCTs can integrate many different submodels that potentially use different modeling types (e.g., physics-based, data-driven, rule-based) that necessitate different strategies and approaches for generating credibility evidence. ISCT submodels can include those for the medical device, the patient, the interaction of the device and patient, generating virtual patients, clinical decision making and simulating an intervention (e.g., device implantation), and translating acute physics-based simulation outputs to health-related clinical outcomes (e.g., device safety and/or effectiveness endpoints). Establishing the credibility of each ISCT submodel is challenging, but is nonetheless important because inaccurate output from a single submodel could potentially compromise the credibility of the entire ISCT. The objective of this study is to begin addressing some of these challenges and to identify general strategies for establishing ISCT credibility. Most notably, we propose a hierarchical approach for assessing the credibility of an ISCT that involves systematically gathering credibility evidence for each ISCT submodel in isolation before demonstrating credibility of the full ISCT. Also, following FDA Guidance for assessing computational model credibility, we provide suggestions for ways to clearly describe each of the ISCT submodels and the full ISCT, discuss considerations for performing an ISCT model risk assessment, identify common challenges to demonstrating ISCT credibility, and present strategies for addressing these challenges using our proposed hierarchical approach. Finally, in the Appendix we illustrate the many concepts described here using a hypothetical ISCT example.

## 1 Introduction

**Figure 1:**
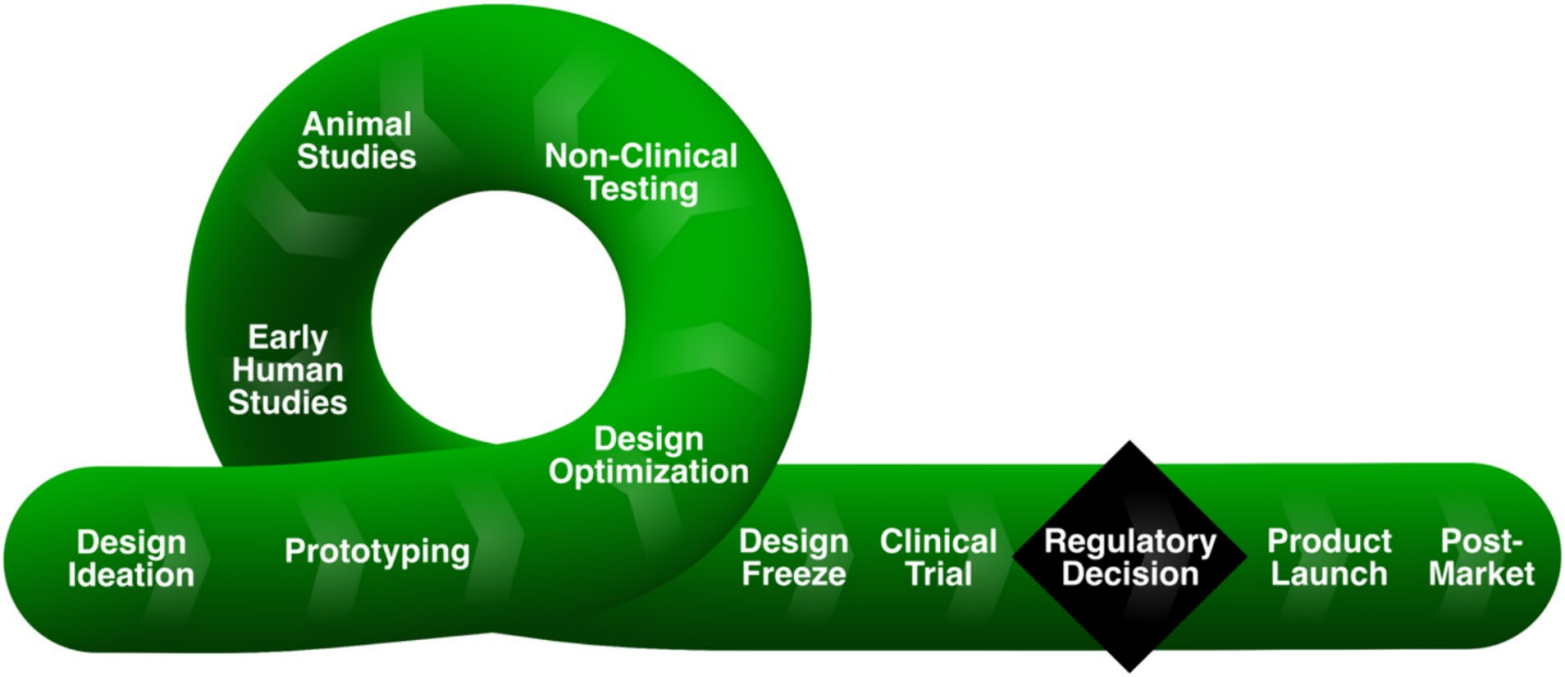
Medical device total product life cycle (TPLC), reprinted from (1) under a CC-BY license. Inspired by and redrawn from Figure 1 in Morrison et al. (2).

Computational models of patients and medical devices can be combined to perform an *in silico* clinical trial (ISCT) to investigate questions related to device safety and/or effectiveness across the total product life cycle (TPLC; Figure 1). In the design stage, an ISCT can be performed to assess device fit or to optimize device design for improved performance. ISCTs can also be used to inform non-clinical bench testing (e.g., by predicting worst-case physiological conditions for the anticipated patient population to justify *in vitro* testing) and animal studies (e.g., to aid in selecting an appropriate animal model). In each of these pre-clinical stages, an ISCT can potentially more quickly inform device design or testing, often based on surrogate quantities that are believed to correlate with device safety and/or effectiveness.

An ISCT can also be used to refine, reduce, or in some cases to completely replace human participants in a clinical trial (3, 4). Generally, refining a clinical trial patient population is known as “enrichment,” in which the goal is to prospectively select study participants that are more likely to respond to a planned intervention (5). An ISCT can likewise be performed to “augment” a clinical trial by incorporating virtual patients to reduce the number of human participants (6, 7). Finally, there is the potential that an ISCT can be performed to replace all human participants in a clinical trial with virtual patients (8, 9). In each case, when the objective is to influence a real human clinical trial, the predictive output from an ISCT should generally be the same as the real trial. For medical devices, this often takes the form of device safety and/or effectiveness outcomes of the intervention or, potentially, surrogate endpoints that are known to strongly correlate with such outcomes.

ISCTs can have many different components, or submodels, including physics-based computational models, data-driven or statistical models, and rule-based models. Physics-based models may include those for the medical device, the patient, and the coupled device-patient. Data-driven or statistical models can be used to generate virtual patient cohorts that are representative of real patients. Data-driven or reduced-order methods can also be used to develop a computationally efficient surrogate model [e.g., (10)] that is trained on the output from physics-based computational modeling and simulation (CM&S) and is then used in lieu of performing numerous computationally expensive physics-based simulations for the ISCT. Rule-based models can be used to emulate clinical decision making or a treatment protocol, for example by prescribing the steps needed to virtually replicate the surgical implantation of a medical device. Finally, because predictions from computational simulations are often in the form of acute, physics-based quantities (stress, strain, pressure, velocity, temperature, etc.), an empirical or data-driven mapping model is often needed to translate the acute predictions to clinical outcomes (e.g., device safety and/or effectiveness endpoints) (11).

ISCTs have many potential benefits to patients, device manufacturers, regulators, and the healthcare system (e.g., reduced costs, expedited time to market, reduced patient risk, increased confidence in device safety and effectiveness) (12). The challenge, however, is that an ISCT is a virtual representation of the real world that needs to be shown to be credible before being relied upon to make decisions that have the potential to cause significant patient harm. In establishing ISCT credibility, it is important to consider the credibility of each submodel that is being used, as inaccurate output from a single submodel could potentially corrupt the entire ISCT.

There are several challenges in establishing the credibility of ISCTs [e.g., see (11)]. Some of the challenges include:

1. Collecting high-quality non-clinical and clinical evidence for validation
2. Use of different modeling types (e.g., physics-based, data-driven, rule-based) for submodels may necessitate different strategies and approaches for generating credibility evidence
3. Demonstrating that non-patient-specific aspects of virtual patients are *“similar, in a precisely defined way, to real patients”* (13)
4. Demonstrating that the virtual treatment emulates the clinical treatment protocol, while accounting for potential procedural variability
5. Establishing and/or validating the mapping model that translates acute physics-based predictions to health-related clinical outcomes (e.g., device safety and/or effectiveness endpoints)

For challenge 5, there are generally two possible scenarios for clinical outcome mapping models. In the first scenario, the mapping relationship is well established, but potentially needs to be rigorously validated with clinical data. In the second, more common, scenario, the relationship may be hypothesized, but it is not well established. In this situation, the mapping model needs to first be developed using clinical data and subsequently validated with additional clinical data, which is difficult and potentially time-consuming. To establish ISCT credibility, each of these challenges has to be overcome.

## 2 Objectives

The objective of this study is to begin addressing some of these challenges and to identify general strategies for establishing the credibility of an ISCT. The study is organized following Appendix 2 (“Reporting Recommendations for CM&S Credibility Assessment in Medical Device Submissions”) of the FDA Guidance “Assessing the Credibility of Computational Modeling and Simulation in Medical Device Submissions” (14), hereafter referred to as the FDA CM&S Credibility Guidance. In Section 3 (Description of Computational Model), we first provide suggestions for ways to clearly describe each of the ISCT submodels and the full ISCT. We then discuss considerations when performing an ISCT model risk assessment in Section 4 (Model Credibility Assessment). Here, we also propose a hierarchical approach for assessing the credibility of an ISCT that involves systematically gathering credibility evidence for each ISCT submodel before demonstrating credibility of the full ISCT. For each part of the credibility assessment, we identify common challenges and present strategies for addressing these. *In doing so, use of the terms “should” and “recommend” are not meant to convey regulatory expectations or guidance, but rather technical considerations based on the experience of the authors*. Finally, in the Appendix we illustrate the many concepts described here using a hypothetical ISCT example. This study complements Pathmanathan et al. (15) that reviews a range of ISCTs published in the literature, discusses how the activities of verification, validation, and uncertainty quantification apply to ISCTs, and provides a high-level workflow that could be used to evaluate an ISCT, including relevant credibility factors.

## 3 Description of computational model

**Figure 2:**
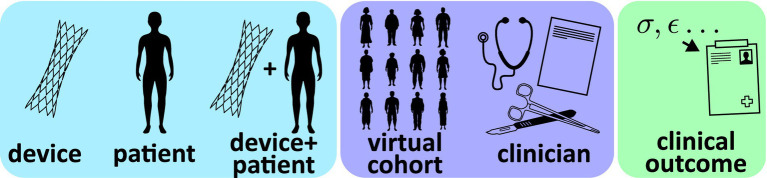
Schematic illustration of possible submodels comprising an ISCT.

As described in the Introduction, ISCTs will often combine different submodels that potentially utilize different types of modeling (physics-based, data-driven, rule-based). To facilitate documentation and credibility assessment, it is important to describe each of the submodels that contribute to an ISCT. As illustrated in Figure 2, most ISCT submodels can generally be classified into one of the following categories:

- *Device model*: typically a mechanistic or physics-based model of the device, including the computational domain, discretization, and associated constitutive relations; specific physics of interest include but are not limited to fluid dynamics, solid mechanics, electromagnetics, optics, acoustics, heat transfer, or combinations thereof
- *Patient model*: typically a mechanistic or physics-based model of the patient (e.g., anatomy or physiology) that will interact with the device, including the boundary conditions, computational domain, discretization, and any constitutive relations
- *Coupled device-patient model*: typically a mechanistic or physics-based model combining the device and patient models, including specific numerical boundary or interface conditions (e.g., contact) used to model interactions between the device and the patient
- *Virtual patient cohort model*: a collection of geometries, material properties, boundary conditions, and other data, either sourced from real patient-level measurements or sampled from statistical distributions, that are used to generate patient-specific or ‘synthetic’ virtual patient models
- *Clinician model*: a model translating clinical decision making and device instructions for use into initial conditions and other inputs for the coupled device-patient model; the model will generally be rule-based or potentially clinician-informed
- *Clinical outcome mapping model*: an empirical or data-driven mapping model (or transfer function) that maps acute physics-based simulation outputs to clinical outcomes (e.g., device safety and/or effectiveness endpoints)

In describing the ISCT submodels, relevant recommendations for non-credibility content from Sections IV–IX of the FDA Guidance “Reporting of Computational Modeling Studies in Medical Device Submissions” (16) may be helpful. For example, the submodel descriptions may include the type of model, modeling assumptions, the governing equations and numerical methods, necessary input data, and quantities of interest that are predicted. Finally, the full ISCT should be described, including a summary of the overall computational workflow and the flow of data among the different submodels.

### 3.1 Device model

The device model description provides details on the computational model used to represent the medical device of interest. Specific details should include the geometry, any assumptions, the type of physics considered, the underlying governing equations, numerical method(s), domain discretization, and the software and solver(s) used. The approaches used for characterizing all inputs to the model should also be described, including the device geometry and any relevant material properties (e.g., mechanical, electromagnetic, thermal, acoustic). A tabular summary of model inputs and predicted quantities of interest can be provided to facilitate understanding of the high-level function of the model.

### 3.2 Patient model

**Figure 3:**
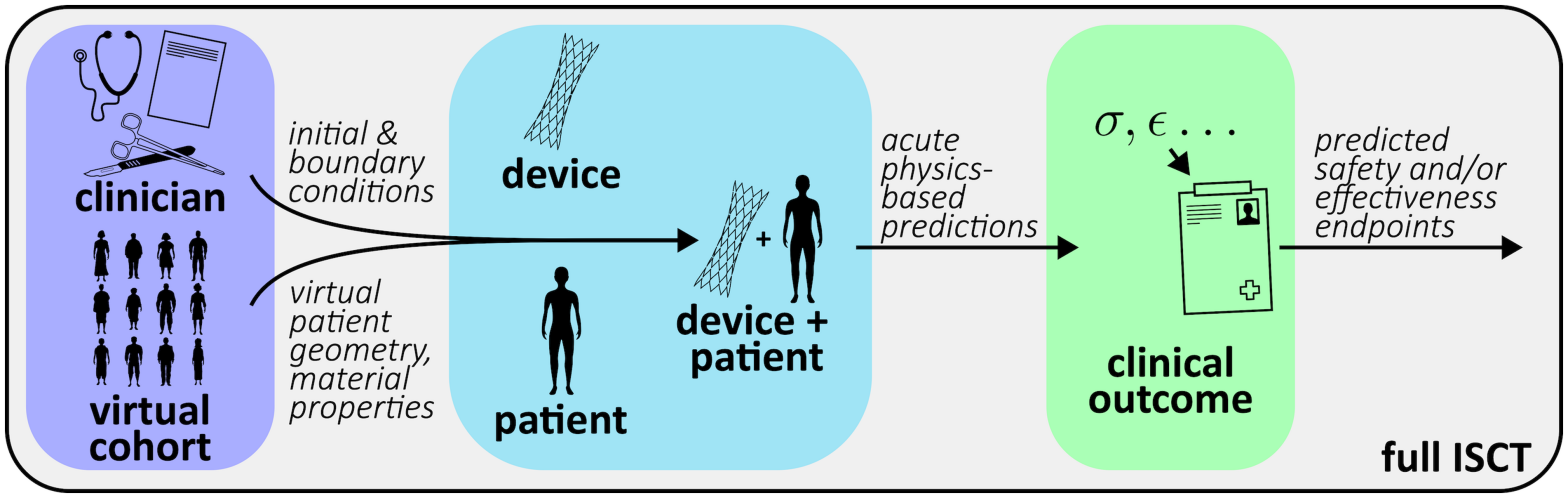
Generic workflow of an ISCT combining submodels from Figure 2.

The patient model is the baseline computational model used to represent virtual patients. Contents of the patient model description should be similar to that provided for the device model description, including any assumptions, the type of physics considered, the underlying governing equations, numerical implementation, domain discretization, and the software and solver(s) used. The approach that is used to represent anatomical geometry should also be described, including whether the geometry is patient-specific, parametric, or otherwise generated. In contrast to the device model that will primarily rely on models of engineering materials (with the exception of bioprosthetic devices), the patient model will often include the use of biological material properties. In most applications, the geometry, material properties, and the initial and boundary conditions of individual patient models in the ISCT will not be fixed but will instead be driven by inputs from the virtual patient cohort models (see Figure 3). Strategies for sourcing and using these data are provided in Sections 3.4. However, to facilitate credibility activities, it may be useful to describe the nominal geometry, material properties, and boundary conditions. As with the device model, a tabular summary of the model inputs and the predicted quantities of interest may be useful.

### 3.3 Coupled device-patient model

The coupled device-patient model is the combination of the device and patient models used to predict the acute performance of the device in terms of physics-based quantities of interest. Predicted performance quantities may be related to safety, effectiveness, or both. The model description should include details on how the device and patient models are coupled, including the specific numerical boundary or interface conditions used (e.g., contact) and whether one- or two-way coupling is performed. If the device is virtually implanted, numerical steps or procedures used to simulate the implantation should be described, including how initial and boundary conditions are driven by the clinician model as applicable (see Section 3.5).

### 3.4 Virtual patient cohort model

The virtual patient cohort model is used to provide input data to the baseline patient model, including patient geometry, material properties, initial and boundary conditions, and any other relevant information. There are generally two approaches to creating virtual patients (17): (i) using patient-level data from real human subjects to create patient-specific models, and (ii) generating “synthetic” patients using population-based statistical information. Both approaches may also be combined to create a virtual patient cohort. For example, the geometry may be based on patient-specific models reconstructed from medical image data, while the material properties are not based on patient-specific information and instead use population-level data. Additionally, the geometry of a healthy or pathologic patient may be morphed based on population-level data to create virtual patients.

The specific approach used for defining each parameter of the virtual patients should be described. When patient-specific information is used to generate virtual patients, the data source and methods used to obtain the information should be summarized. For example, when patient-specific anatomies are reconstructed from medical image data, the imaging modality and resolution should be provided and the approach used to reconstruct the models should be described, potentially including an assessment of the reconstruction accuracy. When a synthetic virtual patient cohort is created, it is important to demonstrate that the synthetic patients are *“similar, in a precisely defined way, to real patients”* (13). This may be the case, for example, when the patient anatomies are created using statistical methods (e.g., statistical shape modeling, patient-averaging) or generative artificial intelligence. Similarly, the methods used to specify any other non-patient-specific information used to inform the virtual cohort should be described and the values justified.

### 3.5 Clinician model

The clinician model translates clinical decision making and device instructions for use into a form such that the treatment can be simulated with the coupled device-patient model (see Section 3.3). Note that device instructions for use and clinical best practices often include both quantitative and qualitative information. Quantitative information will generally be more easily converted to computational algorithms, although there may be challenges with estimating relevant input quantities for virtual patients. To address qualitative instructions or recommendations, there are three primary possibilities: (i) inclusion of a real clinician or clinicians in the process to inform decisions driving the virtual treatments, (ii) development of rules or algorithms to mimic clinical decision making based on information such as pre-procedural virtual patient characteristics and/or model predictions, or (iii) a combination of (i) and (ii).

The clinician model also provides a mechanism for considering procedural variability that is characteristic of a real human clinical trial. That is, given a single virtual patient and a set of device instructions for use, multiple clinicians could be consulted to inform virtual treatments and the varied responses considered separately, or the model could automatically predict multiple treatment approaches that a clinician would reasonably follow (e.g., device size selection, number of devices placed). Additionally, procedural variability could be implemented by introducing stochasticity in the device placement location and orientation or other important characteristics of the specific treatment of interest.

The clinician model description should summarize the approach used, including details regarding the type of model implemented, whether the model is deterministic or stochastic, the necessary input parameters and their respective sources, and the specific strategy for considering clinical decision making, especially when qualitative statements in device instructions for use are involved. Consultation with clinicians should also be described when relevant. Depending on the complexity of the approach, a summary schematic or table may be helpful to illustrate the overall workflow.

### 3.6 Clinical outcome mapping model

As described in the Introduction, for an ISCT to influence a real human clinical trial, final ISCT simulations will often need to predict the safety and effectiveness endpoints used in the real trial. However, clinical trial endpoints are typically in the form of health-related outcomes, not acute physics-based quantities that are common outputs from modeling and simulation. Accordingly, an empirical correlation or mapping model is needed to convert acute physics-based predictions to health-related clinical outcomes (e.g., pain, hospitalization, mortality). In practice, developing this connection may be one of the most difficult challenges to performing an ISCT (11). Two general possibilities exist: (i) the mapping relationship between acute physics-based quantities and clinical outcomes has been previously established, or (ii) hypotheses for the relationship exist, but the specific relationship needs to be both established and validated.

The mapping model description should identify which scenario above is applicable and summarize the overall approach that is used. The type of mapping should also be described. Possibilities include the use of simple empirical relationships or the use of data-driven (e.g., machine learning) techniques. Further recommendations for establishing and validating the mapping model are provided in Sections 4.1 and 4.5.6.

### 3.7 Full ISCT

The full ISCT combines all of the submodels to predict clinical quantities of interest or outcomes that are used to influence the real human clinical trial. Given the potential complexity of ISCTs, it may be helpful to provide a summary of the overall computational workflow using a concise textual description with an accompanying schematic illustrating the flow of primary input and output quantities among the various submodels (e.g., Figure 3). The summary can also highlight key physics-based predictions and how these are translated to final predictions of clinical endpoints.

## 4 Model credibility assessment

### 4.1 Summary of hierarchical credibility assessment approach

As described in the Introduction, because an ISCT is a virtual representation of the real world, it needs to be shown to be credible before being relied upon to make decisions that have the potential to cause significant patient harm. Establishing ISCT credibility is challenging, however, due to the potential complexity of the ISCT framework and the combination of different models and approaches. One strategy to establishing ISCT credibility is to use a hierarchical, or building block, approach that is common in the verification, validation, and uncertainty quantification (VVUQ) community for complex engineering systems (18, 19). In this approach, a complex system is broken down into component subsystems, and VVUQ activities are performed for each to establish their credibility. This helps to ensure that the overall model generates the “*right answer for the right reasons*” (20, 21).

A similar hierarchical credibility assessment approach can be used for ISCTs by breaking the ISCT framework down into its constituent submodels. VVUQ activities can then be performed for each submodel to demonstrate credibility. In this way, we can assess the accuracy of the output from each submodel, which is important to consider given that inaccurate output from a single submodel could potentially corrupt the ISCT results.

We propose a hierarchical approach for ISCT credibility assessment that involves systematically collecting credibility evidence for each ISCT submodel in isolation before demonstrating credibility of the full ISCT. The FDA CM&S Credibility Guidance (14) defines *credibility evidence* as *“any evidence that could support the credibility of a computational model”* and it recommends organizing the evidence into one of eight categories (Table 1). The categories include both traditional verification and validation activities as well as other approaches to establishing model credibility such as reliance on model calibration evidence, observations of emergent model behavior, and scientific evidence or rationale supporting model plausibility.

**Table 1:** Credibility evidence categories from the FDA CM&S Credibility Guidance (14).

No.	Category
①	Code verification results
②	Model calibration evidence
③	Bench test validation results
④	*In vivo* validation results
⑤	Population-based validation results
⑥	Emergent model behavior
⑦	Model plausibility evidence
⑧	Calculation verification/UQ results using COU simulations

The first step is to identify the types of evidence that will be collected to establish the credibility of each ISCT submodel and the full ISCT. Table 2 summarizes the credibility evidence categories that are most relevant for each. Importantly, note that Table 2 does not imply that all of the listed activities should be performed to establish ISCT credibility. The specific activities that are needed to demonstrate credibility will generally depend on the model risk assessment.

**Table 2:** Evidence categories from Table 1 that are most relevant for establishing the credibility of the various ISCT submodels and the full ISCT.

ISCT submodel	Credibility evidence category*							
	Code verification ①^†^	Model calibration ②	Bench test validation ③	*In vivo* validation ④	Population-based validation ⑤	Emergent model behavior ⑥	Model plausibility ⑦	Calc. Ver./UQ using COU simulations ⑧
Device model								
Patient model								
Coupled device-patient model								
Virtual patient cohort model								
Clinician model								
Clinical outcome mapping model								
Full ISCT								

*This table does not imply that all of the listed activities should be performed to establish ISCT credibility. The specific activities that are needed to demonstrate credibility will generally depend on the model risk assessment.^†^Code verification includes both software quality assurance (SQA) and numerical code verification (NCV) (22). Dark gray indicates both SQA and NCV are applicable. Light gray indicates only SQA applies.

For ISCTs, we recommend methodically considering the evidence needs for each submodel in the context of these categories and formulating plans for collecting the associated evidence. As summarized in Table 3, evidence can be collected throughout the medical device TPLC. As evidence is collected, the credibility of each submodel can be assessed to determine whether further model refinements or evidence collection activities are needed before proceeding with the next stages of the ISCT.

**Table 3:** Stages of the medical device total product life cycle (TPLC) when ISCT credibility evidence may be collected (indicated with dark gray).

ISCT submodel	Medical device TPLC stage							
	Design ideation	Device prototyping	Design optimization	Non-clinical bench testing	Animal studies	Early feasibility clinical study	Traditional feasibility clinical study	Pivotal clinical study
Device model								
Patient model								
Coupled device-patient model								
Virtual patient cohort model								
Clinician model								
Clinical outcome mapping model								
Full ISCT								

One unique challenge to establishing ISCT credibility is the need for gathering *in vivo* validation evidence. The challenge is particularly difficult for novel devices for which relevant retrospective clinical data do not exist and, thus, new clinical data needs to be acquired. To address this challenge, similar to the framework proposed by Bodner and Kaul (11), one strategy is to use a staged approach to collecting credibility evidence that incorporates data that are routinely collected as part of the TPLC for moderate- and high-risk devices. As summarized in Table 3, we recommend developing plans for measuring relevant quantities of interest during any *in vivo* studies that are to be performed. By carefully planning these prospective studies, the data can be used to support hierarchical ISCT credibility assessment. For example, data from animal studies may be useful to support credibility of the patient model and the coupled device-patient model. Data from early or traditional feasibility studies in humans can also be used to validate these two submodels as well as the virtual patient cohort model and the clinician model. The clinical outcome mapping model can likewise be established (if unknown) and validated during early or traditional feasibility clinical studies. In this way, each of the ISCT submodels can be validated in isolation. The full ISCT may then be validated using clinical data from a traditional feasibility study, or from the first part of a staged pivotal clinical trial. Such a well-planned and staged approach provides an opportunity to establish ISCT credibility using routinely collected evidence. In documenting the overall process, tabular summaries of the credibility activities may be useful (e.g., Tables 2, 3).

In the following, we describe general considerations for establishing the credibility of an ISCT. We begin by introducing the risk-informed framework for assessing the credibility of modeling and simulation from ASME V&V 40–2018 (22) and the FDA CM&S Credibility Guidance (14). We then describe the different sources of hierarchical evidence that could be used to establish ISCT credibility with some domain-specific examples.

### 4.2 Question of interest

ASME V&V 40–2018 defines the *question of interest* as *“the specific question, decision, or concern that is being addressed”* (22). The FDA CM&S Credibility Guidance provides additional recommendations in formulating the question of interest. Specifically, it is not helpful to formulate a question that is extremely broad (e.g., “should the device be approved?”), nor should the question directly concern the computational model. Rather, it is most useful to formulate the question of interest in terms of a decision that is to be informed *“using the computational model and potentially other sources of information, but nothing more”* (14).

There are many possibilities for formulating a question of interest for an ISCT. As explained in the Introduction, an ISCT can be performed in either the pre-clinical or clinical stages of the medical device total product life cycle. For pre-clinical ISCTs, there are numerous potential questions of interest for device design, guiding non-clinical bench testing, or informing animal studies. At the clinical stage, an ISCT can be used to enrich, augment, or to completely replace human subjects. In these latter cases, an ISCT question of interest will generally be formulated in terms of a decision concerning the real human clinical trial, often related to device safety and/or effectiveness outcomes of an intervention. For example, for an ISCT used to enrich a clinical trial patient population, a possible question of interest could be “what enriched patient enrollment criteria should be used to prospectively exclude patients who are less likely to respond favorably to the intervention?” When an ISCT is performed to augment or completely replace all human subjects with virtual patients, the question of interest will generally be defined in terms of the device safety and/or effectiveness decision(s) that would normally be informed by the real trial.

### 4.3 Context of use

The next step in the framework is to define the model *context of use* (COU), which is *“a statement that defines the specific role and scope of the computational model used to address the question of interest”* (22). The COU should generally consist of a precise description of the computational model and the specific outputs that will contribute to informing the question of interest. For pre-clinical ISCTs, there are numerous possible COUs. In contrast, for ISCTs that intend to influence a real human clinical trial, the COU should generally describe how the ISCT will refine, reduce, or replace human subjects. Additionally, the specific physics-based outputs from the ISCT should be described and how they will be used to inform decision making, potentially through the use of a clinical outcome mapping model. Care should be taken in formulating the COU, as it provides the specific context in which model credibility will be assessed.

### 4.4 Model risk assessment

**Figure 4:**
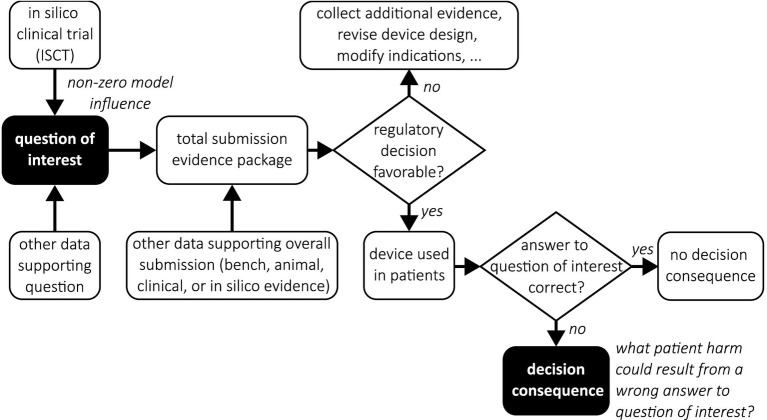
Decision tree flowchart illustrating the use of ISCT evidence to inform a proposed clinical trial in a medical device regulatory submission. Filled boxes emphasize the question of interest and the associated decision consequence.

Given the question of interest and the COU, the next step is to perform a model risk assessment. *Model risk* is defined as *“the possibility that the computational model and the simulation results may lead to an incorrect decision that would lead to an adverse outcome”* (22). Model risk is assessed by considering two independent factors: *model influence* and *decision consequence*. Evaluating model risk for an ISCT is potentially challenging due to the possible existence of multiple decisions that are being made (e.g., regulatory decision, decisions concerning the clinical trial). To simplify the process, as illustrated in Figure 4, one strategy is to begin with the question of interest and trace how the ISCT results will inform any decisions that are to be made. This helps to facilitate the identification of potential decision consequences for incorporation into the model risk assessment.

#### 4.4.1 Model influence

*Model influence* is *“the contribution of the computational model relative to other contributing evidence in making a decision”* (22). As illustrated in Figure 4, both the ISCT and other potential data may be used to inform the question of interest. Assessing model influence essentially consists of evaluating the weight given to the ISCT evidence relative to any other sources of data that will be used to make the decision concerning the question of interest. As recommended by ASME V&V 40–2018 and illustrated in Table 4, model influence can be evaluated in a qualitative fashion by constructing a gradation of possible model influence scenarios, ranging from no-influence to high-influence, and then choosing the scenario that best corresponds to the approach that is used to inform the question of interest.

**Table 4:** Example gradation of possible model influence situations for an ISCT.

Model influence	Description
None	No influence. ISCT outputs are used as supplemental information with no direct influence on the decision.
Low	ISCT outputs have a *minor influence* on the decision. Other evidence is primarily used in making the decision.
Low-medium	ISCT outputs have a *moderate influence* on the decision. ISCT evidence is *weighted less heavily* than other supporting evidence in making the decision.
Medium	ISCT outputs have a *moderate influence* on the decision. ISCT evidence is *weighted equally* with other supporting evidence in making in the decision.
Medium-high	ISCT outputs have a *moderate influence* on the decision. ISCT evidence is *weighted more heavily* than other supporting evidence in making the decision.
High	ISCT outputs have a *dominant influence* on the decision. *No other supporting evidence* is used in making the decision.

#### 4.4.2 Decision consequence

*Decision consequence* is *“the significance of an adverse outcome resulting from an incorrect decision”* (22). The FDA CM&S Credibility Guidance (14) further emphasizes that:

“It is important to note that the decision consequence is the potential outcome of the overall decision that is to be made by answering the question of interest, outside of the scope of the computational model and irrespective of how modeling is used. That is, decision consequence should consider the question of interest, but should not consider the COU of the model”.

For pre-clinical ISCTs, the decision consequence can vary widely. For an ISCT performed to influence a real human clinical trial, decision consequence will often be in the form of potential patient harm in the event that an incorrect decision is made regarding the question of interest.

**Figure 5:**
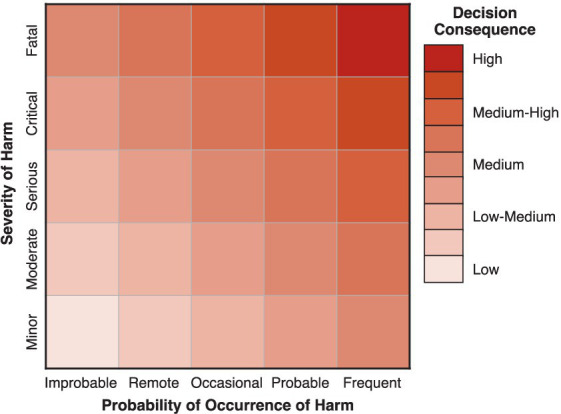
Example of a 5 × 5 matrix that may be used to combine estimates of *severity* and *probability of occurrence* of patient harm for assessing *decision consequence*.

As suggested by ASME V&V 40–2018 and recommended by the FDA CM&S Credibility Guidance, decision consequence can be assessed using principles of risk management such as those detailed in ISO 14971:2019 (23) and ISO/TR 24971:2020 (24). In this case, the general approach consists of identifying possible *hazardous situations* that could result from making an incorrect decision concerning the question of interest and then estimating both the *severity* and *probability of occurrence* of patient harm for each identified *hazard*. Estimates of *severity* and *probability of occurrence* of harm can be categorized using qualitative or semi-quantitative scales (e.g., Tables 5, 6 that are based on examples from ISO/TR 24971:2020). If available, prior adverse event reports for similar devices may be useful in making these estimates (14). The potential consequence of each hazard can then be evaluated by combining the severity and probability estimates as illustrated in Figure 5, which is known as a “risk matrix” in the framework of ISO 14971:2019. The overall decision consequence is subsequently assessed by considering the totality of individual consequences for all potential hazards that could lead to patient harm.

**Table 5:** Example of five qualitative levels for characterizing the potential *severity* of patient harm.

Severity of harm	Description
Minor	Minor patient injury that does not require any medical treatment.
Moderate	Potential for serious patient injury that requires clinical management and/or monitoring.
Serious	Severe pain and/or potential for severe patient injury that requires a medical procedure and risk of complication.
Critical	Severe patient injury that is life threatening.
Fatal	Sudden patient death.

**Table 6:** Example of five quantitative levels for characterizing the *probability of occurrence* of patient harm.

Probability of occurrence of harm (*P*_harm_)	Probability range (or Description)*
Improbable	*P*_harm_ < 0.01%
Remote	0.01% ≤ *P*_harm_ < 0.1%
Occasional	0.1% ≤ *P*_harm_ < 1%
Probable	1% ≤ *P*_harm_ < 10%
Frequent	*P*_harm_ ≥ 10%

*Depending on the specific application and availability of reliable data, either qualitative or quantitative levels can be used (24).

#### 4.4.3 Model risk

**Figure 6:**
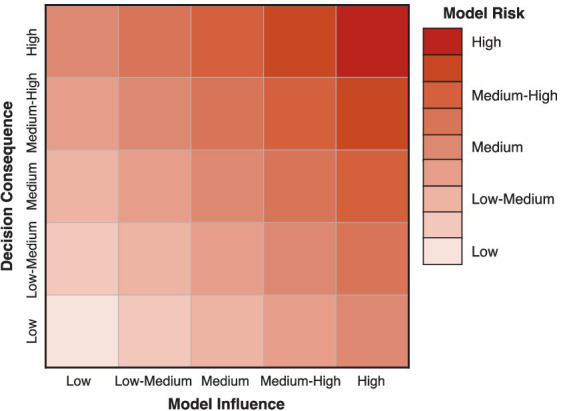
Example of a 5 × 5 matrix that may be used to combine estimates of *model influence* and *decision consequence* for assessing *model risk*.

Following ASME V&V 40–2018, *model risk* is assessed by combining the estimates of *model influence* and *decision consequence*, often in the form of a model risk matrix as illustrated in Figure 6. In this way, we can estimate a semi-quantitative level for the model risk that can be used to guide the planning of VVUQ activities that are performed to establish model credibility. In general, the rigor of VVUQ activities should be such that the overall credibility of a model (or, in this case, an ISCT) should be commensurate with the model risk (22).

### 4.5 Credibility evidence

After performing model risk assessment, activities should be planned and executed to generate credibility evidence. Depending on the specific use of the ISCT, the range, quantity, and quality of evidence needed will vary. In the following sections, we discuss the importance of demonstrating credibility of each of the six ISCT submodels (Sections 3.1—3.6) and the full ISCT (Section 3.7). We also identify the primary evidence categories from the FDA CM&S Credibility Guidance (14) that are most relevant for supporting the credibility of each ISCT submodel and the challenges associated with collecting this evidence. As summarized in Table 2, note that code verification applies to all ISCT submodels. In general, code verification consists of two distinct activities (22): (i) software quality assurance (SQA) and (ii) numerical code verification. Software quality assurance is performed to *“ensure that the software is functioning correctly and produces repeatable results on a specified computer resource in a specified software environment”* (22). SQA code verification, thus, generally applies to all ISCT submodels. Numerical code verification, however, only applies to first principles-based (e.g., mechanistic or physics-based) models in which mathematical governing equations are coded in software and are solved numerically. This code verification activity is performed to ensure that the mathematical governing equations, which often take the form of ordinary or partial differential equations, are implemented and solved correctly (25). Thus, numerical code verification will not generally apply to the virtual patient cohort model, the clinician model, or the clinical outcome mapping model, unless the models incorporate first principles-based components.

Additionally, note that the FDA CM&S Credibility Guidance (14) primarily addresses establishing the credibility of first principles-based models. Although some evidence categories are relevant for establishing the general credibility of model components that are not strictly first principles-based, such as model plausibility evidence supporting input parameter estimation or population-based evidence supporting virtual patient cohort generation, assessing the credibility of other types of models (e.g., data-driven, rule-based) is out of scope and is not addressed here.

#### 4.5.1 Device model

##### 4.5.1.1 Significance

Establishing credibility of the device model in isolation builds confidence that the model accurately simulates device physics relevant to the question of interest. For example, for a computational solid mechanics model of a cardiovascular implant, gathering bench test validation evidence can demonstrate that the chosen governing equation formulation, constitutive laws, and material parameters are appropriate. Moreover, credibility evidence supporting the device model can typically be generated under highly controlled laboratory conditions well in advance of the collection of clinical data. Assessing credibility of the device model in isolation also facilitates the performance of code and calculation verification activities in the absence of complex interactions with a patient model.

##### 4.5.1.2 Primary evidence categories

- *Category* ① - Code verification results. *Relevance*: Performing numerical code verification for the device model provides reassurance that the governing equations and the associated constitutive laws are properly implemented in the software. This is accomplished by comparing numerical results with benchmark solutions, such as exact analytical solutions of the governing equations or those generated using the method of manufactured solutions (MMS) (18, 25). *Challenges*: Rigorous numerical code verification necessitates knowledge of the precise equations that are being solved by the simulation software (26). In some situations, the precise form of the governing equations or constitutive laws may not be known—for example, if proprietary models are used in commercial software. Communication with the software vendor or developers can be helpful in these situations.
- *Category* ② - Model calibration evidence. *Relevance*: The governing equations that are solved for the device model often include empirical parameters that need to be calibrated (e.g., constitutive laws with material model parameters). Evidence of robust calibration demonstrating a good fit to the data using calibrated empirical parameters under conditions that are relevant for the COU provides confidence that the parameter values are appropriate. *Challenges*: In some cases, it can be challenging to calibrate empirical parameters under the full range of conditions that are relevant for the COU. For example, calibration of material model parameters for nickel-titanium (nitinol) shape memory alloys in computational solid mechanics generally necessitate experimental data for a representative material lot from which devices are manufactured under both tensile and compressive loading conditions, which can be difficult to acquire. In addition, calibration is ideally performed using experimental data for the primary quantities of interest. Otherwise, if calibration is performed based on global or integrated quantities alone (e.g., force-displacement in solid mechanics), there is no guarantee that the calibrated parameters will yield accurate predictions of local quantities of interest such as stress or strain (27). This can be challenging if the primary quantities of interest are difficult to measure, and in some cases it is not feasible. For example, acquiring detailed measurements of turbulence in blood flow in complex devices for calibration of computational fluid dynamics (CFD) turbulence model parameters is not practically feasible. Thus, default model parameters calibrated using historical benchmark data (e.g., turbulent boundary layer measurements in a wind tunnel) are often used. In these cases, when it is not possible to calibrate empirical parameters under realistic conditions using data for the primary quantities of interest, validation of the device ISCT submodel becomes even more critical.
- *Category* ③ - Bench test validation results. *Relevance*: Bench testing provides opportunities to simplify boundary and loading conditions relevant to the analysis of the device while minimizing potentially confounding factors from neighboring tissues and/or physiological systems. Bench experiments also provide an ideal environment for reducing uncertainty and variability in validation experiments. For example, validation evidence to support a computational solid mechanics model for a device could be collected by subjecting the device to a physiologically relevant loading mode while measuring physics-based quantities of interest. If considerable disagreement between the device model and experimental observations is observed under these ideal conditions, the device model should be revised and validation activities repeated before incorporating the device model into the overall ISCT. *Challenges*: Access to physical devices is needed to collect bench test evidence to support the device model. Accordingly, this type of evidence cannot be collected until prototype devices are manufactured. Performing bench test validation also necessitates access to experimental facilities and test equipment.

#### 4.5.2 Patient model

##### 4.5.2.1 Significance

Similar to Section 4.5.1, establishing credibility of the patient model in isolation builds confidence that the patient model accurately simulates the physics for the patient anatomy or physiology of interest. For example, for a computational solid mechanics model of a biological tissue, gathering benchtop validation evidence (e.g., using cadaveric specimens), animal data, or human clinical evidence can help demonstrate that the chosen governing equations, constitutive models, and material parameters are appropriate. Examining the patient model in isolation likewise facilitates the performance of code and calculation verification activities in the absence of complex interactions with a device model.

##### 4.5.2.2 Primary evidence categories

- *Category* ① - Code verification results. *Relevance*: Numerical code verification of the isolated patient model may be performed to confirm the implementation of constitutive relationships and the governing equations that are specifically relevant to biological tissues and/or physiological phenomena. *Challenges*: In contrast to the device model that will often consider engineering materials with well-established physical behavior, the patient model may have more complex nonlinear constitutive relationships and potentially custom numerical implementations. The patient model may also need to capture both healthy and pathological disease states, further complicating the governing equations and the associated numerical code verification exercises.
- *Category* ② - Model calibration evidence. *Relevance*: Fully defining all parameters for the patient model may be difficult in practice. For example, some model parameters may not be phenomenological or cannot be directly measured. In such cases, calibration activities can be performed to tune unknown model parameter values such that the model output matches experimental data. As described in the FDA CM&S Credibility Guidance (14), model calibration evidence is not a substitute for validation, but it can still contribute to establishing model credibility. *Challenges*: Challenges highlighted for category ② evidence for the device model similarly apply here. In general, patient models can have numerous parameters. Thus, calibration of the patient model involves careful consideration, especially if many parameters are being calibrated simultaneously.
- *Category* ③ - Bench test validation results. *Relevance*: Validation performed by comparing with bench testing provides the opportunity to simplify boundary and loading conditions relevant to the biological tissues and systems of interest while minimizing potentially confounding factors associated with *in vivo* conditions. Bench test conditions also facilitate measuring quantities of interest more directly and with higher accuracy than is generally possible clinically. *Challenges*: Test articles representative of the patient anatomy of interest (e.g., cadaver specimens or realistic phantoms) are needed for bench tests supporting validation of the patient model. Developing bench tests that closely reproduce *in vivo* conditions may be difficult, lowering the applicability of the evidence.
- *Category* ④ - *In vivo* validation results. *Relevance*: *In vivo* conditions provide the most realistic opportunity for validating the patient model. *Challenges*: *In vivo* conditions are often complicated. Collecting adequate patient-level information to fully define patient-matched models is challenging, and often only geometric information is readily available. For example, directly measuring patient-specific material properties may not be possible, necessitating either parameter estimation or separate calibration activities (i.e., category ② evidence) that introduce additional uncertainty in the validation comparison. Acquiring data from patients in the population of interest may also be difficult, especially for devices treating rare diseases. *In vivo* measurements of quantities of interest may necessitate acquisition of data beyond standard clinical practice that could increase patient risk (e.g., extending treatment times or increasing radiation exposure).

#### 4.5.3 Coupled device-patient model

##### 4.5.3.1 Significance

Even when the device and patient models are demonstrated to be highly credible in isolation, there is no guarantee that the coupled device-patient model will accurately predict device performance. Accordingly, additional credibility activities help to demonstrate that predictions from the combined model are reliable.

##### 4.5.3.2 Primary evidence categories

- *Category* ① - Code verification results. *Relevance*: Coupling the device and patient models will often involve the implementation of new boundary or interface conditions. Additional numerical code verification may be needed to demonstrate that these conditions are properly implemented if prior verification activities do not provide adequate coverage. *Challenges*: Performing numerical code verification that considers all governing equations involved in the coupled device-patient model may be challenging. If numerical code verification has been performed for both the device and patient models, one potential option is to narrowly scope the verification activities to focus on the coupled interaction part of the model using simplified test cases or relevant benchmark problems.
- *Category* ② - Model calibration evidence. *Relevance*: Similar to the individual device and patient models, some aspects of the coupled model may be difficult to measure directly. For example, for solid mechanics simulations of device placement and follow-on performance in a patient anatomy, characterizing friction coefficients may be difficult without relying on surrogate measurements. In such scenarios, calibration evidence can support model credibility. Again, follow-on validation evidence is critical when key model parameters rely on calibration. *Challenges*: Challenges are largely the same as those expressed in the model calibration bullets of Sections 4.5.1 and 4.5.2.
- *Category* ③ - Bench test validation results. *Relevance*: Like Sections 4.5.1 and 4.5.2, bench testing provides an opportunity for characterizing the accuracy of the coupled device-patient model under well-controlled and idealized laboratory conditions. For example, for devices for which the performance relies critically on patient geometry, bench experiments can be performed using physical models based on patient medical imaging data. Similarly, for devices that strongly interact with the patient model (e.g., device-soft tissue interaction), bench testing can be performed using a realistic phantom or cadaver specimen. *Challenges*: Challenges highlighted previously for category ③ evidence apply here. The applicability of bench test validation activities relative to *in vivo* conditions should be assessed. If the bench test conditions are not representative of *in vivo* conditions, the results of the validation activity do not guarantee that the model will accurately predict *in vivo* device performance. Even so, a model that performs poorly at predicting device performance under well-controlled bench test conditions will unlikely yield accurate predictions under much more complex *in vivo* conditions. For this reason, bench test validation of the coupled device-patient model should be considered when there are questions about important physical interactions.
- *Category* ④ - *In vivo* validation results. *Relevance*: *In vivo* measurements collected using animals or humans provide the most applicable evidence to support the credibility of the coupled device-patient model. *Challenges*: Physics-based predictions of some quantities of interest from the coupled device-patient model may be difficult to validate due to the inability to measure those quantities under *in vivo* conditions, which may necessitate relying on measurements of surrogate quantities. Animal and human data will also likely be more costly and time-consuming to acquire compared to bench evidence.
- *Category* ⑦ - Model plausibility. *Relevance*: If the coupled interaction and interface conditions are simple and any associated parameters are well-established or are easily measured, model plausibility evidence may be provided to establish the credibility of the coupled device-patient model. This will usually take the form of a thoughtful justification of any underlying assumptions, the coupled nature of the interaction (e.g., weak coupling), the form of the coupling conditions, and any model parameters. *Challenges*: Some strongly coupled phenomena (e.g., some fluid–structure interaction problems) cannot be accurately solved by weakly coupling two separate models (e.g., device and patient models). For such problems, advanced numerical methods and solution schemes are required to ensure an accurate coupled solution. In this case, model plausibility evidence may not be sufficient to establish the credibility of the coupled device-patient model.

#### 4.5.4 Virtual patient cohort model

##### 4.5.4.1 Significance

ISCTs rely on evidence generated from simulations of virtual patient cohorts. As described in Section 3.4, there are generally two approaches to creating virtual patients (17): (i) using patient-level data from real human subjects to create patient-specific models, and (ii) generating “synthetic” patients using population-based statistical information. Both approaches may also be combined to create a virtual patient cohort.

Establishing credibility of the virtual patient cohort model in isolation helps to ensure that the ISCT results are correct for the right reasons, including because the virtual population is representative of real patients. For any patient-specific information that is used to generate virtual patients (e.g., anatomies or material properties), the data source and methods used to obtain the information should be summarized. Credibility can be further established by assessing the accuracy of the patient-specific models or information–e.g., the accuracy of the geometric reconstruction of medical image data (see Section 4.5.2).

For any synthetic components of virtual patient cohort models, it is important to demonstrate that non-patient-specific information is *“similar, in a precisely defined way, to real patients”* (13). For example, if a synthetic cohort is generated by uniformly sampling the parametric space describing patient anatomies or material properties, statistical comparisons of the distributions of the virtual cohort and real patient data can demonstrate credibility. In doing so, it may be important to consider the potential existence of correlations between some parameters. For example, patients with enlarged anatomies may also tend to have material properties within a specific range. The comparison of synthetic patient and real patient data may potentially include either model input parameters (e.g., geometry, material properties) or clinical outcomes. A rigorous comparison of all input parameters provides strong credibility evidence for the virtual patient cohort model in isolation. While comparing results from the full ISCT (either physics-based predictions or clinical outcomes) with real clinical data can provide some reassurance of the credibility of the virtual patient cohort model, it does not guarantee that the model is credible because the other submodels contribute to the ISCT predictions. If possible, it is thus generally best to establish the credibility of the virtual patient cohort model in isolation.

##### 4.5.4.2 Primary evidence categories

- *Category* ① - Code verification results. *Relevance*: SQA code verification may be performed for the virtual patient cohort model. Numerical code verification, however, will not generally apply unless there are first principles-based components to the model. *Challenges*: Virtual patient cohort models may often use open-source or custom software. If the software is developed without adherence to SQA practices, performing SQA code verification may be difficult.
- *Category* ⑤ - Population-based validation results. *Relevance*: A statistical comparison of virtual cohort characteristics with population-level data adds credibility that the virtual cohort is representative of the intended patient population. *Challenges*: Population-level data may not be available for all of the relevant patient characteristics. Additionally, some characteristics predicted by the model may not be measurable in patients and, thus, cannot be directly validated. Capturing correlations among characteristics may also be challenging.
- *Category* ⑦ - Model plausibility evidence. *Relevance*: Model plausibility evidence is scientific rationale that can support the virtual patient cohort model in multiple ways. For example, if information on parameter distributions is unavailable, parameter ranges could be based on limited data reported in the literature and on expert clinical judgment. Scientific rationale may be used in this situation to support the chosen ranges. If virtual cohorts are designed to represent a specific sub-population where there is a dearth of information—for example, edge-cases, rare or extreme anatomies, or a rare disease—then along with any available literature data, scientific rationale may be used to support the virtual cohort model. *Challenges*: Category ⑤ challenges also apply here.

#### 4.5.5 Clinician model

##### 4.5.5.1 Significance

As described in Section 3.5, the clinician model converts clinical decision making and device instructions for use into a form (e.g., an algorithm) that can be used by the coupled device-patient model to simulate device performance in virtual patients. Accurately simulating the intervention is critical for ISCT predictions to mimic those of the real human clinical trial. For example, even if the device, patient, and coupled device-patient models have been rigorously verified and validated, if clinical decision making is not properly accounted for in the clinician model, ISCT predictions could significantly differ from real clinical trial observations. Accordingly, evidence should be provided to support the credibility of the clinician model.

##### 4.5.5.2 Primary evidence categories

- *Category* ① - Code verification results. *Relevance*: SQA code verification may be performed for the clinician model. Numerical code verification, however, will not generally apply unless there are first principles-based components to the model. *Challenges*: Challenges described for SQA code verification of the virtual patient cohort model (Section 4.5.4) also apply here.
- *Category* ④ - *In vivo* validation results. *Relevance*: Similar to Section 4.5.3, subject-level results from animal or human studies can be used to support correct implementation of the clinician model. For example, the clinician model could be used to blindly predict the placement location and orientation of a device in a given subject, and the predictions could be compared to post-procedure clinical observations. *Challenges*: Collecting subject-level evidence to support validation of the clinician model involves careful planning to ensure that the acquired measurements and observations are adequate. For new devices, the number of cases available for collecting treatment data prior to the pivotal clinical trial may be limited.
- *Category* ⑦ - Model plausibility evidence. *Relevance*: To replicate clinical practice, the clinician model needs to follow device instructions for use and existing clinical guidelines. Evidence demonstrating that these details are fully captured in the clinician model may be used to support credibility. *Challenges*: As mentioned in Section 3.5, device instructions for use and clinical guidelines typically include both qualitative and quantitative information. Implementing the former in the clinician model may involve establishing relationships between physics-based model parameters and qualitative instructions.

#### 4.5.6 Clinical outcome mapping model

##### 4.5.6.1 Significance

A crucial step for ISCTs to reduce, refine, or replace clinical trials is demonstrating that they can predict health-related clinical outcomes of an intervention, which are often in the form of device safety or effectiveness endpoints. Because CM&S predictions are usually in terms of acute physics-based quantities, an empirical correlation or mapping model is needed to convert physics-based predictions to clinical endpoints such as pain, hospitalization, or mortality. This is further complicated by the time course of clinical follow-up that can range from several months to years, which may necessitate the need to account for physiological adaption or remodeling. Moreover, while physics-based predictions will generally be continuous, clinical outcomes are often binary or categorical. Evidence is therefore needed to both develop and validate the mapping function. Potential evidence sources include previous clinical knowledge from the literature, retrospective clinical data for the disease state of interest or related treatments (e.g., similar devices), and prospective clinical data collected specifically for the device from early or traditional feasibility studies. In the latter case, the prospective clinical trial may need to be specifically designed to capture important quantities of interest that would not be normally acquired otherwise. The overall credibility of the mapping function depends critically on the strength of the correlation between acute physics-based quantities and clinical outcomes, and on the availability of appropriate supporting evidence.

##### 4.5.6.2 Primary evidence categories

- *Category* ① - Code verification results. *Relevance*: SQA code verification may be performed for the clinical outcome mapping model. Numerical code verification, however, will not generally apply unless there are first principles-based components to the model. *Challenges*: Challenges described for SQA code verification of the virtual patient cohort model (Section 4.5.4) also apply here.
- *Category* ② - Model calibration evidence. *Relevance*: Clinical follow-up of animal or human subjects can be used to develop correlations between physics-based predictions and clinical endpoints. Demonstrating a strong correlation can help to establish the credibility of the mapping model. *Challenges*: In the absence of existing clinical knowledge or follow-up data from retrospective clinical studies, the relationship between acute physics-based quantities and clinical outcomes may necessitate performing new clinical studies. Early or traditional feasibility studies using the device may provide sufficient evidence to develop the mapping model given an initial hypothesis.
- *Category* ④ - *In vivo* validation results. *Relevance*: For newly developed mapping functions, subject-level information on patient follow-up from retrospective or new clinical studies can be used to validate the link between physics-based quantities and clinical endpoints. *Challenges*: Validating the mapping function may necessitate performing new clinical studies. Additionally, it may not be possible to acquire the physics-based quantities of interest in the clinical study that are used in the mapping function.
- *Category* ⑤ - Population-based validation results. *Relevance*: Statistically comparing population-level data of the correlation between physics-based quantities and clinical outcomes observed in real patients can increase the credibility of the mapping function. *Challenges*: Population-level data may not be available for mapping all physics-based ISCT quantities of interest and clinical endpoints.
- *Category* ⑦ - Model plausibility evidence. *Relevance*: Before initiating an ISCT, the high-level plausibility of relying on physics-based quantities to predict clinical outcomes for the application of interest should be carefully assessed. Scientific rationale can be documented based on literature studies, existing clinical data, subject-matter expertise, and other evidence sources. *Challenges*: For new devices, direct evidence supporting the clinical outcome mapping model will not exist, although evidence from related devices may provide some information.

#### 4.5.7 Full ISCT

##### 4.8.7.1 Significance

The full ISCT combines all submodels to predict clinical quantities of interest that are used to influence (or replace) the real human clinical trial. If rigorous hierarchical credibility assessment is performed to verify and validate all of the ISCT submodels in isolation, it may be possible to forego rigorously validating the full ISCT. This, however, will depend on the model risk assessment and the adequacy of the credibility evidence for all of the ISCT submodels. If the credibility of some ISCT submodels cannot be adequately established in isolation, then it becomes critical to validate the full ISCT by comparing with clinical data from a traditional feasibility study or from the first part of a staged pivotal clinical trial (see Table 3). In either case, it is important to consider calculation verification and uncertainty quantification (UQ) of the COU simulations performed using the full ISCT (Category ⑧ evidence in Table 1).

##### 4.5.7.2 Primary evidence categories

- *Category* ④ - *In vivo* validation results. *Relevance*: Subject-level data from clinical studies can be used to establish the credibility of the full ISCT. The clinical data could be from either a prospectively planned trial or from a retrospective study. In the latter case, the data should be independent of those used to calibrate any of the ISCT submodels. If hierarchical credibility assessment was not performed for all ISCT submodels, care should be taken to examine the credibility of any unvalidated submodels as part of the final validation of the full ISCT. For example, if the credibility of the coupled device-patient and clinician submodels were not assessed in isolation, intermediate quantities of interest (e.g., post-procedure location and deformation of an implantable device) could be acquired in this final clinical validation study. *Challenges*: Data acquired in retrospective studies may be inadequate or have limited applicability. Performing a prospective clinical study to validate the full ISCT and any unvalidated submodels involves careful planning. Also, it may not be possible to clinically measure quantities that are needed to validate physics-based predictions from some submodels.
- *Category* ⑤ - Population-based validation results. *Relevance*: Similar to category ④, population-level data from clinical studies can be used to establish the credibility of the full ISCT, and potentially some unvalidated submodels (e.g., virtual patient cohort model, clinical outcome mapping model). *Challenges*: Category ④ challenges also apply here.
- *Category* ⑧ - Calculation verification/UQ results using COU simulations. *Relevance*: After validating the ISCT submodels and the full ISCT, the ISCT context of use (COU) simulations are performed that are used to address the question of interest (e.g., to enrich or augment the real human clinical trial). At this stage, it may be important to quantify uncertainty in the final ISCT predictions by performing calculation verification for the applicable submodels (i.e., the device, patient, and coupled device-patient models) and performing UQ. Alternatively, if prior credibility activities address calculation verification and/or UQ and are applicable to the final ISCT COU simulations, then this information can be used to increase the credibility of the ISCT results. *Challenges*: It is extremely computationally expensive to perform calculation verification and/or UQ for all virtual patients in an ISCT. As discussed by Pathmanathan et al. (15), there are several potential strategies for addressing this challenge (e.g., performing analyses on expected worst-case virtual patients).

### 4.6 Adequacy assessment

For a given COU, adequacy assessment is *“the process of evaluating the credibility evidence in support of a computational model, together with any other relevant information, possibly including results from the COU simulations, and making a determination on whether the evidence is sufficient considering the model risk”* (14). Detailed recommendations for adequacy assessment are provided in Section VI.D of the FDA CM&S Credibility Guidance (14). In brief, adequacy assessment may occur either prospectively or following collection of credibility evidence (i.e., post-study). In both cases, the totality of the credibility evidence is reviewed to consider whether it is (or would be) sufficient to *“support using the model for the COU given the risk assessment”* (14). For an ISCT, adequacy assessment involves considering the planned or achieved credibility of each individual submodel, as well as the full ISCT, and judging the collective adequacy.

A critical exercise for ISCTs will be defining new credibility factors for non-traditional evidence. Credibility factors are *“elements of the process used to establish the credibility of the computational model for a COU”* (14, 22). ASME V&V 40–2018 provides credibility factors for verification, validation, and applicability of traditional credibility evidence gathered through bench testing. Aldieri et al. (28) provide an example of using these credibility factors for an ISCT. The FDA CM&S Credibility Guidance (14) provides some recommendations and examples for defining new credibility factors for non-traditional evidence sources (e.g., model calibration, emergent model behavior, model plausibility). Recent work by Pathmanathan et al. (15), Bischoff et al. (29), and Galappaththige et al. (30) provides further recommendations and examples.

After defining credibility factors, credibility goals are proposed (in preparation for prospective adequacy assessment) or the achieved credibility levels are summarized (to support post-study adequacy assessment). The overall model credibility is then evaluated relative to the model risk. In this adequacy assessment step, the objective is to justify that the overall model credibility is commensurate with model risk. Given the potential complexity of ISCTs, it may be helpful to create a tabular or graphical summary of the credibility factors for each of the ISCT submodels and summarize the credibility goals or credibility levels achieved to facilitate the adequacy assessment activity [e.g., see Figure 3 in (14)]. For post-study adequacy assessment, final ISCT predictions for the COU may also be considered, especially if the question of interest contains a safety or decision threshold (e.g., fatigue safety factor) (14). In performing adequacy assessment, rationale may be needed whenever the credibility of a particular submodel or submodels is lacking–for example, if several of the credibility goals are not achieved. Overall, assessing adequacy involves making a careful decision concerning the credibility evidence with a thoughtful justification using all available information (14).

For ISCTs that augment or replace a real human clinical trial, one unique challenge for adequacy assessment is considering the extent to which the ISCT addresses all of the clinical trial endpoints. Ideally, when the goal is to replace human subjects in a medical device clinical trial, an ISCT should predict the same safety and effectiveness endpoints as the real trial. In practice, however, it may be challenging to predict all endpoints with an ISCT, even through the use of clinical outcome mapping models. For example, multifactorial adverse events or subjective endpoints such as pain and other patient reported outcomes may be especially difficult to predict. It is, thus, important to consider whether all endpoints can be fully addressed with an ISCT.

In general, patient sample size in a medical device clinical trial is driven by the statistical power needed to demonstrate a *“reasonable assurance of safety and effectiveness”* (31). Therefore, if an ISCT is used to replace patients in a clinical trial, it is imperative that all endpoints are adequately addressed to avoid underpowering the study. In the extreme case of when an ISCT is designed to replace all human subjects, this means that either (i) the ISCT should predict all trial endpoints, or (ii) endpoints not predicted by the ISCT are addressed with other evidence such as non-clinical data (e.g., cadaver, animal, or bench testing). A similar situation exists for an ISCT designed to augment a clinical trial by replacing some patients, except that the reduced human clinical trial can also contribute to the totality of the endpoint evidence. In this case, the combination of evidence from real patients, the ISCT, and other sources (e.g., non-clinical testing) should ideally provide the same endpoint information that would be obtained from a traditional clinical trial.

In practice, obtaining the same endpoint information from an ISCT (and other sources) as a traditional clinical trial may be challenging. Because the sample size needed to power specific endpoints can vary (31), one strategy for tackling this challenge is to separately consider the sample size needed for each endpoint. In this way, if a specific trial endpoint cannot be accurately modeled and is not included in the ISCT (e.g., see (32)), the adequacy of the reduced sample size for that endpoint can be independently assessed. Using this approach provides an opportunity to combine evidence from multiple sources (real patients, ISCT, non-clinical testing) to adequately address all clinical trial endpoints. Further research and examples of implementing this proposed strategy are, however, needed.

## 5 Summary and conclusions

An ISCT is a virtual representation of the real world that needs to be shown to be credible before being relied upon to make decisions that have the potential to cause patient harm. There are many challenges to establishing ISCT credibility. In this study, we begin to address some of these challenges and to identify general strategies for overcoming them.

We first discuss how ISCTs often combine different submodels that potentially utilize different modeling modalities (physics-based, data-driven, rule-based, empirical). These submodels can include those for the medical device, the patient, the interaction of the device and patient, generating virtual patients, clinical decision making and simulating an intervention (e.g., device implantation), and translating acute physics-based simulation outputs to health-related clinical outcomes (e.g., device safety and/or effectiveness endpoints). We explore the different possibilities for each submodel, and we provide suggestions for ways to clearly describe each and the full ISCT following the FDA CM&S Reporting Guidance (16) and the FDA CM&S Credibility Guidance (14). We then discuss considerations for performing an ISCT model risk assessment, including recommendations for how to identify potential decision consequences through the use of a decision tree flowchart. Notably, we also propose a hierarchical approach for assessing the credibility of an ISCT that involves systematically gathering credibility evidence for each ISCT submodel in isolation before demonstrating credibility of the full ISCT. In this way, we can evaluate the credibility of each submodel, which is important given that inaccurate output from a single submodel could potentially compromise the credibility of the entire ISCT. This helps to ensure that the overall model (i.e., the full ISCT) generates the *“right answer for the right reasons”* (20, 21). Using the credibility evidence categories from the FDA CM&S Credibility Guidance, we then identify the possible evidence that may be used to establish the credibility of each ISCT submodel and the full ISCT. We also discuss how the evidence can be strategically gathered throughout the medical device total product life cycle. For each submodel, we describe general considerations for establishing credibility using the most relevant types of evidence and present some domain-specific examples. In each case, we identify common challenges and present strategies for addressing these. Finally, we discuss how to assess the adequacy of the credibility evidence and one unique challenge for ISCTs that augment or replace a real human clinical trial.

To summarize, some of the major challenges to establishing ISCT credibility include:

- *Performing model risk assessment*: Evaluating model risk for an ISCT is challenging due to the possible existence of multiple decisions that are being made (e.g., regulatory decision, decisions concerning the clinical trial). To simplify the process, in Section 4.4 (Model Risk Assessment) we recommend using a decision tree flowchart that begins with the question of interest and traces how the ISCT results will inform any decisions that are to be made. This helps to identify potential decision consequences in the model risk assessment.
- *Evaluating hybrid models*: Use of hybrid models that incorporate both physics-based and data-driven components in ISCTs necessitates different strategies and approaches for generating credibility evidence and complicates the overall credibility assessment. Here we follow the FDA CM&S Credibility Guidance that applies to first principles-based models (e.g., mechanistic or physics-based) but is not applicable for data-driven models such as machine learning or artificial intelligence. Although some evidence categories from the FDA CM&S Credibility Guidance are relevant for establishing the general credibility of some non-physics-based models (e.g., population-based evidence supporting virtual patient cohort generation), assessing the credibility of any data-driven modeling is not addressed here. Data-driven modeling is likely to become widely used for several different ISCT submodels, including the virtual patient cohort model, clinician model, and the clinical outcome mapping model. Unfortunately, there is not an established approach for assessing the credibility of hybrid computer models that combine physics-based and data-driven components. Future work is, thus, needed to establish a harmonized credibility assessment framework for hybrid modeling.
- *Performing hierarchical validation*: Acquiring validation evidence for all ISCT submodels is potentially costly and time-consuming, requiring careful planning. To address this challenge, in Section 4.5 (Credibility Evidence) we recommend using a hierarchical approach to credibility assessment that involves strategically gathering validation evidence from activities that are routinely performed as part of the medical device total product life cycle. As summarized in Table 3, this involves developing plans for acquiring validation data during any non-clinical or clinical studies that are to be performed. By carefully planning prospective studies, the data can be used to support hierarchical ISCT credibility assessment. This is especially important for planning costly clinical studies so that validation data are collected efficiently (i.e., to mitigate or reduce the need for conducting separate clinical studies to support ISCT validation). For example, data from early or traditional feasibility studies in humans may be used to validate the patient model, the coupled device-patient model, the virtual patient cohort model, and the clinician model. The clinical outcome mapping model can likewise be established (if unknown) and validated during early or traditional feasibility clinical studies. In this way, each of the ISCT submodels can be validated in isolation. The full ISCT may then be validated using clinical data from a traditional feasibility study, or from the first part of a staged pivotal clinical trial. Such a well-planned staged approach provides an opportunity to establish ISCT credibility using routinely collected evidence.
- *Developing and validating clinical outcome mapping models*: A crucial step for ISCTs is demonstrating that they can predict health-related clinical outcomes of an intervention, which are often in the form of device safety or effectiveness endpoints. Because CM&S predictions are usually in terms of acute physics-based quantities, an empirical correlation or mapping model is needed to convert physics-based predictions to clinical endpoints such as pain, hospitalization, or mortality. This is further complicated by the time course of clinical follow-up that can range from several months to years, which may necessitate the need to account for physiological adaption or remodeling. Moreover, while physics-based predictions will generally be continuous, clinical outcomes are often binary or categorical. Evidence is therefore needed to both develop and validate the mapping function(s). In practice, developing this connection may be one of the most difficult challenges to performing an ISCT (11). In Section 4.5 (Credibility Evidence), as part of our proposed hierarchical credibility assessment approach, we provide several suggestions for ways to overcome this challenge and to acquire the evidence needed to establish and validate the clinical outcome mapping model.
- *Defining ISCT credibility factors*: A critical exercise for ISCTs will be defining new credibility factors for non-traditional evidence. ASME V&V 40–2018 provides credibility factors for verification, validation, and applicability of traditional credibility evidence gathered through bench testing, which have been applied to an ISCT (28). The FDA CM&S Credibility Guidance (14) provides some recommendations and examples for defining new credibility factors for non-traditional evidence sources (e.g., model calibration, emergent model behavior, model plausibility). Recent work by Pathmanathan et al. (15), Bischoff et al. (29), and Galappaththige et al. (30) provides further recommendations and examples. Even so, there is not an established common set of credibility factors for ISCTs. Future work is, thus, needed to establish credibility factors for non-traditional evidence sources, including those used to assess the credibility of hybrid or data-driven models.
- *Performing adequacy assessment*: For ISCTs that augment or replace a real human clinical trial, one unique challenge for adequacy assessment is considering the extent to which the ISCT addresses all of the clinical trial endpoints. Ideally, when the goal is to replace human subjects in a medical device clinical trial, an ISCT should predict the same safety and effectiveness endpoints as the real trial. In practice, however, it may be challenging to predict all endpoints with an ISCT, even through the use of clinical outcome mapping models. For example, multifactorial adverse events or subjective endpoints such as pain and other patient reported outcomes may be especially difficult to predict. To avoid underpowering the clinical study, it is, thus, important to consider whether all endpoints can be fully addressed when using an ISCT to replace patients. In Section 4.6 (Adequacy Assessment), we discuss one strategy for tackling this challenge that involves separately considering the sample size needed for each endpoint. In this way, if a specific trial endpoint cannot be accurately modeled and is not included in the ISCT, the adequacy of the reduced sample size for that endpoint can be independently assessed. Using this approach provides an opportunity to combine evidence from multiple sources (real patients, ISCT, non-clinical testing) to adequately address all clinical trial endpoints. Further research and examples of implementing this proposed strategy are, however, needed.

Depending on the model risk, not all of the aforementioned challenges need to be overcome to successfully use an ISCT for a specific application. But, if ISCTs are to realize their full potential in the future, additional work is needed to address each of these challenges.

## Data availability statement

The raw data supporting the conclusions of this article will be made available by the authors, without undue reservation.

## Ethics statement

The studies involving humans that are described in the Appendix were performed under Institutional Review Board (IRB) approval from Boston Children’s Hospital and Washington University. The studies were conducted in accordance with the local legislation and institutional requirements. The participants provided their written informed consent to participate in this study.

## Author contributions

KA: Writing – review & editing, Writing – original draft. TB: Writing – review & editing, Writing – original draft. AP: Writing – review & editing, Writing – original draft. JY: Writing – review & editing. SK: Writing – review & editing. CC: Writing – review & editing. SP: Writing – review & editing. PP: Writing – review & editing. DH: Writing – review & editing. SL: Writing – review & editing, Writing – original draft. BC: Writing – review & editing, Writing – original draft.
