# Supplementary material for: Toward trustworthy medical device in silico clinical trials: a hierarchical framework for establishing credibility and strategies for overcoming key challenges
Source: Front Med (Lausanne). 2024 Aug 12;11:1433372. doi: 10.3389/fmed.2024.1433372 (PMC11346031; doi:10.3389/fmed.2024.1433372)
Supplement: Supplementary file 1 [file Presentation_1.pdf]

Appendix

Hierarchical Credibility Assessment Example: Hypothetical ISCT of a Generic Edge-to-Edge Mitral Valve Repair Device

Here we present an example of the proposed framework for the hierarchical credibility assessment of ISCTs. In this example, a hypothetical ISCT is performed to provide supporting evidence in defining enrollment criteria for a clinical trial of a generic edge-to-edge mitral valve repair device. In addition to ISCT evidence, other information will be used to define the trial enrollment criteria, including pre-clinical experience in animal studies, enrollment criteria for clinical trials performed for other similar devices, and clinical judgement. The overall strategy consists of defining baseline enrollment criteria based on these other sources of information and then using the results of an ISCT to refine (i.e., “enrich”) the criteria to prospectively exclude patients who are less likely to respond favorably to the intervention. In this hypothetical example, the ISCT only informs enrollment criteria refinement; it does not influence any other aspects of the clinical trial. In the event that the real human clinical trial successfully demonstrates that the device is safe and effective, the device would be indicated for the same patient population studied in the trial.

Our goal is to provide a hypothetical example to illustrate the proposed hierarchical credibility assessment framework for ISCTs following the FDA CM&S Credibility Guidance (1). We first provide example descriptions of the various submodels that comprise the ISCT. We then present abbreviated examples of an ISCT *question of interest* and *context of use* (COU), followed by a hypothetical model risk assessment. Finally, we describe the types of evidence that could be provided to demonstrate the credibility of the hypothetical ISCT. Importantly, we do not present *credibility factors* for the types of evidence or evaluate the adequacy of such evidence. ASME V&V 40-2018 (2) provides credibility factors for verification, validation, and applicability of traditional credibility evidence gathered through bench testing. A critical exercise for ISCTs will be defining new credibility factors for non-traditional evidence sources. The FDA CM&S Credibility Guidance (1) provides some recommendations and examples for defining such credibility factors. Recent work by Pathmanathan et al. (3), Bischoff et al. (4), and Galappaththige et al. (5) provides additional recommendations and examples. The types of credibility evidence described here are provided as possible examples. Any modeling approaches and associated input parameters or boundary conditions are purely hypothetical and do not represent an accepted approach. The execution of the hypothetical ISCT is also not the focus of the present example and results are, thus, not reported.

An edge-to-edge mitral valve repair device was chosen for this example based on the interest and experience of the authors. The inclusion of this example is not intended to imply the current or near-term feasibility of using an ISCT to influence a mitral valve repair device clinical trial. Mitral valve disease, especially secondary mitral regurgitation, is complex and many aspects of the disease remain poorly understood by the clinical community, which has led to diverging results even in randomized controlled clinical trials (6). One critical challenge is the lack of understanding of the correlation between acute device performance and long-term clinical outcomes, which fundamentally hinders the use of an ISCT for this application (and others). Additionally, the complete set of information needed to generate and validate an ISCT for this or any other type of mitral valve repair device is not available at this time, and approaches to conducting an ISCT for this technology for regulatory purposes have not been established. This mitral valve repair device example is, thus, intended only to illustrate the

proposed hierarchical ISCT credibility assessment framework. The inclusion of this example should not be interpreted as an indication of FDA acceptance of ISCTs for mitral valve edge-to-edge repair technologies, or that the methods, results, or justifications would be sufficient for these devices within a regulatory context.

A1. Description of Computational Model

A hypothetical ISCT is performed for a generic transcatheter edge-to-edge mitral valve repair device using a combination of (i) physics-based modeling to simulate device and patient structural mechanics, dynamics, and hemodynamics and (ii) statistical and data-driven modeling to generate a virtual synthetic cohort as inputs to the physics-based patient models. Following the proposed framework (Figure 2 of the main document), the ISCT is organized into six submodels: (i) device model, (ii) patient model, (iii) coupled device-patient model, (iv) virtual patient cohort model, (v) clinician model, and (vi) clinical outcome mapping model (**Figure A1**). Each of these models are addressed in examples that follow.

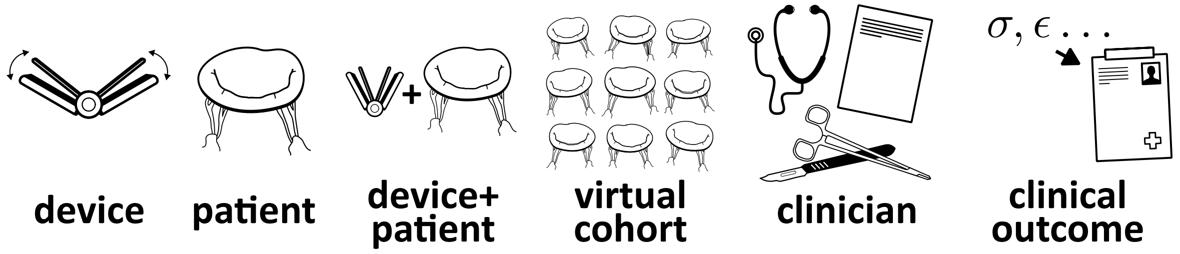

Figure A1: Submodels of the hypothetical ISCT for a generic edge-to-edge mitral valve repair device.

A1.1. Device Model

The device model is a physics-based, finite element (FE) rigid-body dynamics model implemented in Abaqus/Explicit (7). In brief, the model solves the three-dimensional Newton—Euler equations to simulate the motion of the components of a generic mitral valve clipping device (**Figure A2**). The delivery system is not considered. The notional clipping device is symmetrical and consists of two pairs of rotating bodies: two primary “arms” and two “grippers” (**Figure A2-a**). The arms and grippers are both 9 mm long and 4 mm wide, and they have thicknesses of 1.5 mm and 0.5 mm, respectively. The edges of the device are rounded to mitigate numerical contact singularities that could occur when incorporating the model into coupled device-patient simulations (**Figure A2-a**). Stop plates are also implemented to limit leaflet insertion (**Figure A2-a**). A rotational boundary condition about a single axis of rotation is used to constrain the motion of the clip components (**Figure A2b-d**). In addition to the constrained rotational axis, the overall translation and rotation of the clip is controlled using a single rigid body reference node with six degrees of freedom (black dot in **Figure A2-a**). The device is discretized using rigid-body elements and assigned a uniform density.

Limitations and Gaps:

- The device model only considers rigid body motion. The neglect of local device stresses and strains limits the ability of the model to predict device mechanics, and it introduces approximations in the prediction of device performance.
- The model is highly idealized and does not consider components characteristic of a real implantable edge-to-edge repair device including pins, fixation barbs, and polymeric elements.

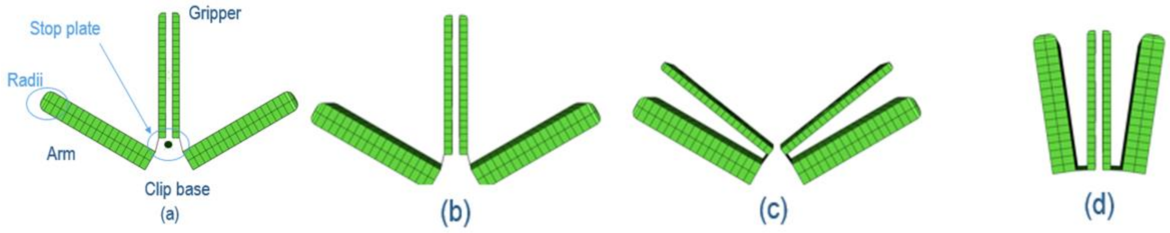

Figure A2: (a) Schematic of the generic mitral valve repair device model in three configurations: (b) fully open, (c) partially closed (leaflet grasping), and (d) fully closed ("locked").

A1.2. Patient Model

The baseline patient model is a multi-physics model performed using a co-simulation between Abaqus/Explicit and Dymola (8) (**Figure A3**). In brief, Abaqus is used to solve the three-dimensional time-resolved Cauchy momentum equations and to simulate valve solid mechanics (**Figure A3-a**). Dymola, a zero-dimensional system-based lumped-parameter tool, is used to estimate a critical boundary condition for the Abaqus simulations, the transmitral pressure difference (**Figure A3-b**). The two solvers are coupled to simulate valve motion during a cardiac cycle (**Figure A3-c)**.

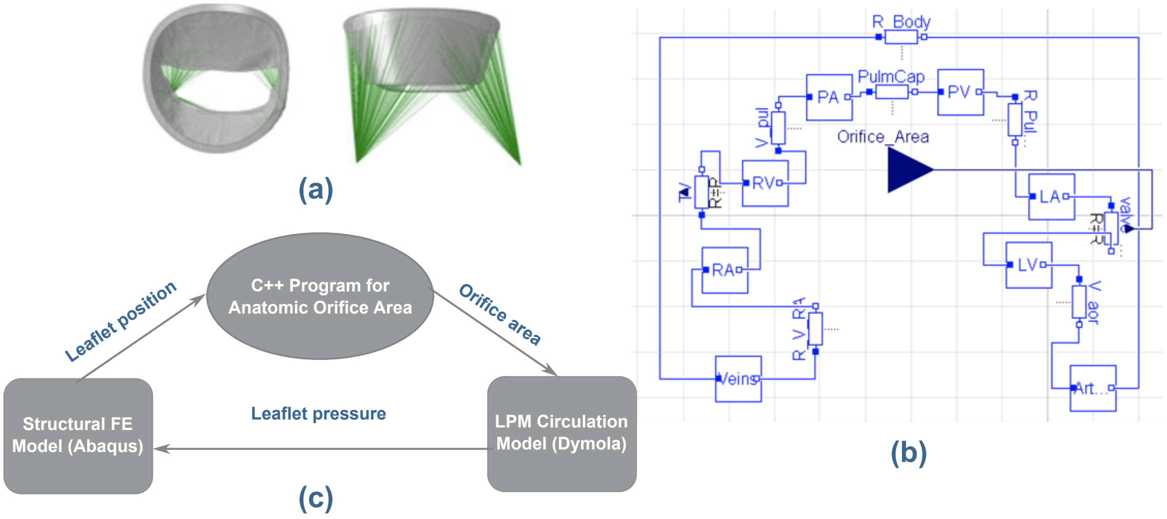

Figure A3: The baseline patient model includes (a) a structural Abaqus finite element model, (b) a Dymola lumped-parameter model, and (c) a co-simulation model that combines the structural and lumped-parameter models to predict the time-varying anatomic orifice area of the mitral valve.

The baseline patient model includes input parameters that are specified to create individual patient models (**Table A1**). Variable input parameters include constitutive model inputs, initial and boundary condition quantities, and key geometric measurements (**Table A1**). By modifying these parameters, the model can be adapted to either approximate a specific real patient or to generate fully synthetic virtual patients.

Mitral Valve Structural Model: The structural model consists of both mitral valve leaflets and idealized chordae (**Figure A3-a**). Although papillary muscles are not modelled, the coordinates of the two papillary-chordae attachment locations are defined (**Figure A3-a** and **Table A1**). Leaflets are modelled with a uniform thickness of approximately 2mm and are discretized using linear, incompatible mode,

hexahedral brick elements. An anisotropic Holzapfel-Ogden constitutive law is used to simulate leaflet tissue. Baseline material constants are calibrated using biaxial human leaflet tensile data from the literature. Chordae are modelled with a uniform cross-sectional area and are discretized using truss elements. The material response of chordae is modelled using a neo-Hookean hyperelastic constitutive law. Chordae insertion points on the leaflets are uniformly distributed along the bottom surface with a uniform density based on observations in literature. No branching of the chordae between the attachment and insertion points is considered. Chordae start at each insertion point and are attached to the nearest papillary-chordae attachment point.

The papillary-chordae attachment locations and the annular edges of the leaflets are prescribed as boundary conditions, both of which can be fixed or time-varying (**Table A1**). General surface-to-surface frictionless contact is defined to account for collisions between opposing leaflets and among nodes within the same leaflet (i.e., self-contact).

Circulatory System Lumped-Parameter Model: Pressure boundary conditions on the valve are predicted using a closed-loop lumped-parameter model (**Figure A3-b**). The model simulates the bulk effects of blood flow within the heart and to and from the circulatory system. The model is comprised of capacitors with active time-varying capacitance to simulate the compliance of ventricular and atrial chambers. Systemic vein, systemic artery, pulmonary vein, and pulmonary artery chambers are simulated as passive capacitors. Resistances are assigned to model flow through the aortic and pulmonary valves and between adjacent chambers. Blood flow initiates in response to the initial pressure difference assigned to the chambers. Flow between the ventricles and atria through the mitral and tricuspid valves is modeled using a Bernoulli-like equation that captures the effect of blood inertance, blood resistance, and time-varying valve orifice area.

Co-simulation Model: Co-simulation between the three-dimensional structural and zero-dimensional lumped-parameter models is performed by coupling the two solutions at a user-defined time interval (**Figure A3-c)**. At each time step, a custom post-processing routine calculates the time-dependent anatomic orifice area of the leaflets given by the structural model and communicates the orifice area to the lumped parameter model (**Figure A3-c**). The lumped parameter model then calculates the instantaneous left ventricular and atrial pressures, and the pressure difference is communicated back to the structural model (**Figure A3-c**). The pressure difference is imposed as a spatially uniform pressure boundary condition on the ventricular side of the mitral valve leaflets.

The coupled mitral valve and circulatory system models are used to predict several acute quantities of interest (**Table A1**). The models are also used to estimate a categorical mitral valve regurgitation severity grade based on model predictions of regurgitant volume (**Table A1**) using a suggested threshold from clinical guidelines (9).

Simulating the Pre-Operative Patient State: The pre-operative state of a patient is simulated using the following steps starting at end diastole:

1. Initial chamber pressures are specified (**Table A1**)
2. The nodes at the annulus and the papillary muscle attachment points are fixed and chordae lengths are adjusted at diastole (lengthened or shortened) by performing an analysis to achieve an approximated target systolic state where most chordae become straight and are under tension. Subsequently, any resulting residual strain in the chordae and leaflets caused by these adjustments are set to zero to approximate the nearly strain-free state at 70% diastole.

3. The co-simulation begins during which pressure is calculated and applied to the leaflets as described above and the annular and attachment node locations that were fixed in step 2 are prescribed to be time-varying
4. Cardiac cycles are simulated until reaching a periodic steady-state response

Table A1: Key Patient Model Inputs and Outputs

Mitral Valve Structural Model Inputs
Leaflet material parameters
Chordae material parameters
Chordae length
Leaflet size and shape
Annulus size, shape, and motion
Chordae-papillary attachment locations and motions

Mitral Valve Structural Model Outputs
Motion and shape of geometric components
Coaptation behavior (height, tenting area, posterior leaflet angle)
Stress and strain in leaflets and chordae
Mitral valve orifice area
Effective orifice area
Regurgitant orifice area and location(s)

Circulatory System Lumped-Parameter Model Inputs
Chamber initial pressures and volumes
Chamber passive compliances
Chamber active compliances
Flow model parameters through mitral and tricuspid valves (resistance, inertance, blood density)
Flow resistances through pulmonary and aortic valves
Flow resistances through chambers (except valves)

Circulatory System Lumped-Parameter Model Outputs
Chamber pressure and volume traces
Left ventricular ejection fraction
Left ventricular systolic and diastolic volumes
Systolic and diastolic transmitral pressure gradient
Regurgitant volume and fraction
Pulmonary veinous waveforms
Mean left atrial pressure

Limitations and Gaps:

- The model only considers mitral valve structural mechanics and simplified circulatory system fluid dynamics. Electrophysiology is not included. The model also neglects any tissue remodeling that may occur in response to changes in patient mechanics or hemodynamics.
- Valve mechanics are complex and rely critically on three-dimensional fluid-structure interactions (10,11). The current model only simulates fluid mechanics using a lumped-parameter approach. The ability of this approach to precisely predict leaflet motion, coaptation behavior, and regurgitation metrics may be limited.
- Accurate mitral valve simulation likewise relies critically on accurate modeling of the geometry of the patient's valve and associated tissues (12). As expressed in literature, "*no clinical imaging modality is capable of visualizing the complete mitral valvular and sub-valvular anatomy with adequate spatial or temporal resolution for accurate computational modeling*" (13). Here, the valve and cardiac anatomy are highly idealized, and chordae tendinea geometry, density, stiffness, and attachment locations are approximated.
- Several key model input parameters such as valve material properties are approximated based on nominal values from literature. Accurately measuring these parameters for patient-specific models or generating representative parameter sets for synthetic patients remains a significant challenge.
- Model outputs are limited to physics-based predictions (**Table A1**).

A1.3. Coupled Device-Patient Model

The coupled device-patient model combines the device and patient models incorporating the discretized valve and clip into the same co-simulation. Specifically, hard frictionless contact is initially prescribed between the device stop plates and the leaflets, and a penalty contact condition is prescribed between the device arms and grippers and the leaflets. The combined model is used to simulate clip deployment (**Figure A4**). First, the clip reference node is translated and rotated (**Table A2**) such that the leaflets are inserted into the clip arms (**Figure A4-a**). Second, the grippers are rotated (**Table A2**) toward the arms to fully grasp the leaflets (**Figure A4-b**). The arms are then rotated inward (**Table A2**) to close the clip (**Figure A4-c**). The rotational magnitudes of the grippers and arms are chosen carefully to ensure adequate contact occurs without excessive leaflet compression and element distortion. Frictionless contact conditions are then converted to bonded (no-slip) to simulate the influence of migration mitigating features of a real device. Finally, boundary conditions on the rigid body reference node are removed, and the clip is allowed to move in six degrees of freedom throughout the co-simulation of the cardiac cycle (**Figure A4-d**).

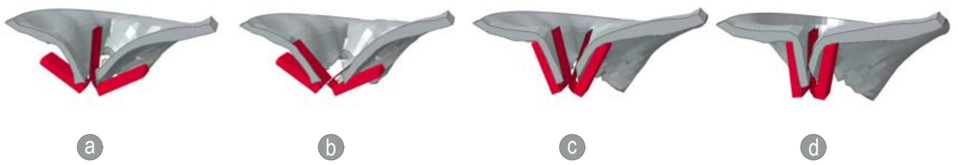

Figure A4: The coupled device-patient model illustrating the four steps used to (a) position, (b-c) deploy, and (d) release the clip.

Table A2: Coupled device-patient model inputs and outputs, in addition to the inputs and outputs listed in **Table A1**.

Inputs
Clip deployment translation and rotation
Rotations of clip grippers and arms

Outputs
Clipping forces and moments prior to clip release
Clipping length, symmetry, and grip pressure
Post-clipping regurgitant orifice location(s)

Limitations and Gaps:

- The coupled model inherits all limitations of the individual device and patient models (see above).
- The chosen contact models idealize interactions between the device and the patient as purely frictionless or bonded. Real contact interactions depend on local surface topology, lubrication, and other aspects not modelled herein. The neglect of barb fixation features in the device model also limits the realism of the contact interactions.
- The model neglects any acute biological effects (e.g., damage) or longer-term remodeling that may occur in the valve in response to device placement.

A1.4. Virtual Patient Cohort Model

The objective of the virtual patient cohort model is to generate a collection of synthetic patients that are representative of real patients that would be enrolled in a real human clinical trial. Note the generation of realistic pathological patients is not trivial, as the influence of each individual input variable on valve

performance may be nonlinear, interactions among input variables are complex, and correlations among input variables are generally unknown. Accordingly, generation of the virtual synthetic patient cohort involves several steps. The approach used here focuses on generating synthetic pathological patients and relies on a combination of physics-based and surrogate modeling to reduce computational costs (**Figure A5**). First, real patient measurements from literature are used to generate probability distributions for key input parameters needed by the baseline patient model (**Table A1**). The probability distributions are then sampled to generate an initial virtual patient cohort (**Figure A5-i**). Patient model finite element simulations are performed using this initial cohort to train a surrogate model for the prediction of primary quantities of interest associated with valve pathology (**Figure A5-ii**). Additional samples are then drawn from the input probability distributions to create a larger virtual cohort (**Figure A5-iii**), and the surrogate model is used to predict quantities of interest for each synthetic patient. Note that, at this stage, the synthetic cohort potentially includes both realistic and unrealistic pathological and healthy patients. To exclude healthy patients, the cohort is filtered based on predefined criteria (**Figure A5-iv**). Finally, a clustering analysis is performed to extract representative pathological patients (**Figure A5-v**) for use in the full ISCT.

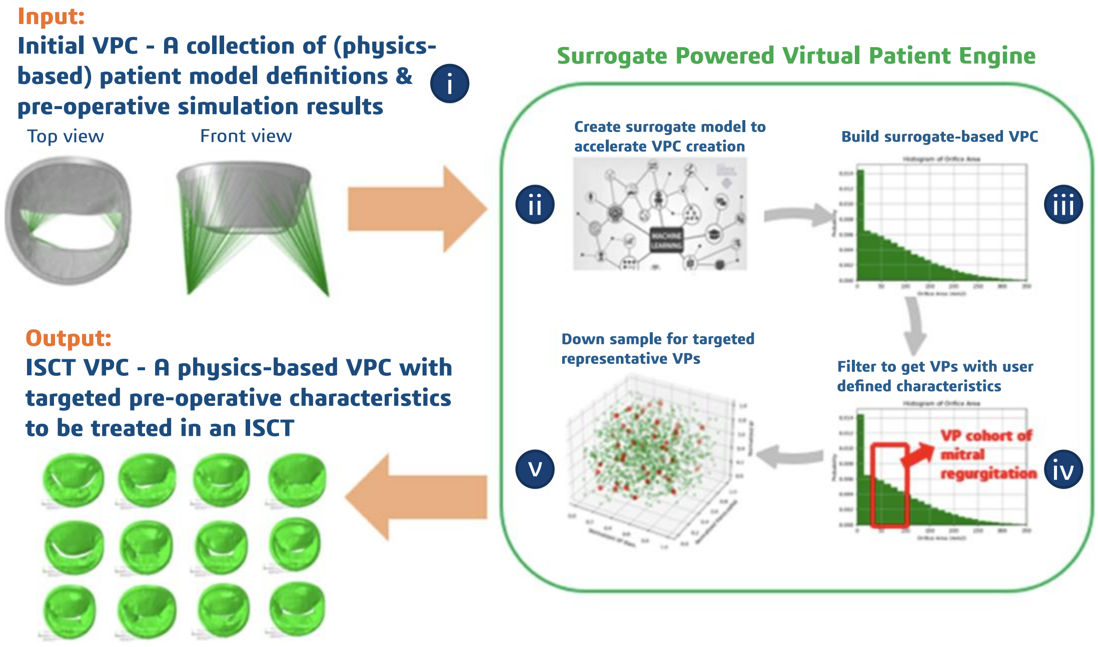

Figure A5: Schematic illustrating an approach for generating a virtual patient cohort with target characteristics. (i) An initial synthetic patient cohort is generated by sampling probability distributions for key model input parameters. (ii) The initial cohort is used to train a surrogate model for predicting quantities of interest related to mitral valve regurgitation. (iii) A second, larger synthetic patient cohort is generated, and the surrogate model is used to predict the severity of regurgitation for each synthetic patient. (iv) The second, larger cohort is filtered to include only pathological patients based on pre-defined criteria. (v) A clustering analysis is performed to extract representative pathological patients for use in the full ISCT.

Limitations and Gaps:

- The proposed filtering process excludes healthy synthetic patients based on predictions of physics-based quantities of interest. However, there is no guarantee that the remaining

synthetic pathological patients are representative of real pathological patients *"in a precisely defined way"* (14). For example, correlations among input parameters are neglected during synthetic patient generation. Accounting for such correlations may be critical to the generation of realistic synthetic patients (15).

A1.5. Clinician Model

The clinician model combines real clinician input and a quantitative decision tree to define clipping boundary conditions for each virtual patient in the ISCT. The model is semi-automated and follows relevant consensus recommendations from clinical literature.

The clinician model proceeds as follows:

- A real clinician examines a set of predictions from the pre-operative patient model and determines where to place a clip along the mitral valve line of coaptation
- An automated procedure is used to combine the device, patient, and coupled device-patient models and to execute the clipping procedure for a first clip
- Based on key predictions from the post-operative physics-based model, a second clip may be placed. In brief, if the predicted transmitral pressure gradient and mitral regurgitation grade fall within predefined ranges, the treatment is complete. If instead the values are outside the target post-treatment ranges, the first clip is repositioned, a second clip is placed, or both.

Limitations and Gaps:

- As implemented, the clinician model does not capture the diversity of all possible treatments, the likely intraprocedural variability among clinicians, or other human factors aspects of the treatment.
- The model uses a single clip size. Real devices feature several sizes to accommodate patient variability.
- The model ensures at least one leaflet is completely seated into the clip. This is not guaranteed during a real clinical procedure.
- The model only places the clip at pre-defined locations between 30% to 70% along the line of coaptation at 10% intervals. Real procedures, however, include a continuum of possible clip placement locations.
- The model does not allow for the same degree of iterative adjustment as in a real intervention.

A1.6. Clinical Outcome Mapping Model

Currently, there is not a widely accepted correlation between acute physics-based quantities of interest and clinical outcomes for edge-to-edge mitral valve repair devices. Accordingly, to predict clinical outcomes using an ISCT, a correlation would first need to be established and then validated.

At a high level, historical data show reducing mitral valve regurgitation via edge-to-edge repair improves heart function and reduces mortality and re-hospitalization in some patients. A mapping function to correlate changes in one or more acute post-procedural clinical measurements (potential quantities of interest) with patient outcomes could be proposed based on clinical studies in the literature. Possible physics-based quantities that may correlate with clinical outcomes include:

- diastolic mitral valve pressure gradient (16)
- residual mitral regurgitation (6,17)

- pulmonary venous waveforms (18)
- ratio of left ventricle end-diastolic volume to effective regurgitant orifice area (proportionate or disproportionate mitral regurgitation) (19)
- decreased left atrial pressure (20)

For the hypothetical ISCT, we propose beginning with the identified correlation between post-operative mean left atrial pressure and mortality identified by El Shaer et al. (20). In brief, the investigators found the patient cohort with post-operative left atrial pressure less than 22 mmHg had higher survival rates compared to the cohort with higher post-operative left atrial pressure (hazard ratio=1.71 [95% CI, 1.10–2.70] P=0.02; see Figure 1 in El Shaer et al. (20) and accompanying text).

Limitations and Gaps:

- As mentioned, there is currently not a widely accepted correlation between acute physics-based quantities and clinical outcomes for edge-to-edge mitral valve repair. The proposed mapping model relies on a correlation recently observed from a clinical study in the literature (20). However, the observation of a correlation in one patient cohort is not guaranteed to be predictive for all patients. The predictive capability of the observed correlation and the associated mapping model should ideally be supported by follow-on independent validation.
- In the study by El Shaer et al. (20), mortality still occurred in the low post-procedural left atrial pressure patient cohort (approximately 25% compared to 40% in the high left atrial pressure patient cohort). Thus, mapping models should consider the strength of underlying correlations and include estimates of the uncertainty in any outcome predictions.

A1.7. Full ISCT

Following the proposed framework (Figure 3 in the main document), the full ISCT combines all of the previously described submodels to predict patient outcomes (**Figure A6**). Specifically, the clinical treatment and virtual cohort models drive physics-based simulations of device placement in virtual patients. Physics-based simulations then predict the post-operative mean left atrial pressure. The clinical outcome mapping model then predicts patient outcomes for the virtual cohort based on the acute, physics-based predictions of post-operative mean left atrial pressure. Finally, the ISCT results are used to inform revisions to the baseline enrollment criteria to prospectively exclude patients who are less likely to respond favorably to the intervention.

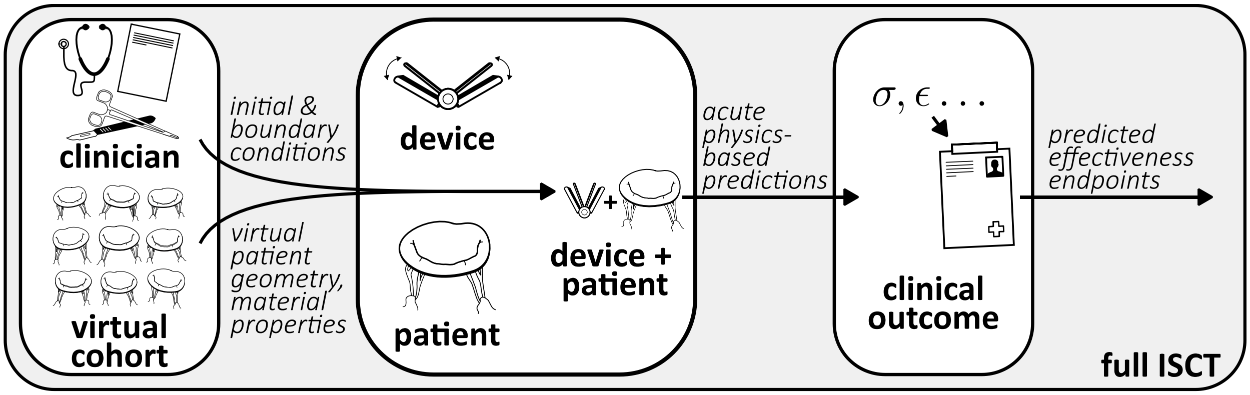

Figure A6: Workflow for the hypothetical ISCT combining submodels from **Figure A1**.

A2. Model Credibility Assessment

A2.1. Summary of Hierarchical Credibility Assessment Approach

The specific activities that are needed to demonstrate credibility of the hypothetical ISCT will depend on the model risk assessment. For most applications that have non-negligible model risk, it is important to demonstrate the credibility of each of the ISCT submodels in some way to ensure that the full ISCT produces the *"right answer for the right reasons"* (21,22). This hypothetical ISCT example follows the proposed hierarchical credibility assessment approach described in the main body of this study. First, the ISCT question of interest and context of use are defined. A model risk assessment is then performed to evaluate the overall ISCT model risk. Given our estimated model risk, we then present credibility evidence. Importantly, note that we do not attempt to provide examples for all of the possible credibility evidence. Rather, we provide several examples of the types of evidence that could be collected for a **subset** of the ISCT submodels that are highlighted in **Table A3**. In doing so, we also do not present credibility factors for the types of evidence or evaluate the adequacy of such evidence. ASME V&V 40-2018 provides credibility factors for verification, validation, and applicability of traditional credibility evidence collected through bench testing. In practice, credibility factors facilitate the systematic review and documentation of the strengths, limitations, and gaps associated with credibility evidence. Although we do not address credibility factors here, we do summarize limitations and gaps for each credibility example. In future work, a critical exercise for ISCTs will be defining new credibility factors for non-traditional evidence sources. The FDA CM&S Credibility Guidance (1) provides some recommendations and examples for defining such credibility factors. Recent work by Pathmanathan et al. (3), Bischoff et al. (4), and Galappaththige et al. (5) provides additional recommendations and examples. The types of credibility evidence described here are provided as possible examples. Any data or comparisons that are presented are purely hypothetical and do not represent an accepted approach for establishing ISCT credibility.

Table A3: Evidence categories from Table 1 of the main document that are most relevant for establishing the credibility of the various ISCT submodels and the full ISCT (gray). Evidence categories that are partially addressed with the hypothetical ISCT of an edge-to-edge mitral valve repair device are highlighted in orange.

	**Credibility Evidence Category***							
**ISCT Submodel**	Code verification ① †	Model calibration ②	Bench test validation ③	In vivo validation ④	Population-based validation ⑤	Emergent model behavior ⑥	Model plausibility ⑦	Calc. Ver./UQ using COU simulations ⑧
Device Model								
Patient Model								
Coupled Device-Patient Model								
Virtual Patient Cohort Model								
Clinician Model								
Clinical Outcome Mapping Model								
**Full ISCT**								

*This table does not imply that all of the listed activities should be performed to establish ISCT credibility. The specific activities that are needed to demonstrate credibility will generally depend on the model risk assessment. †Code verification includes both software quality assurance (SQA) and numerical code verification (NCV) (2). Dark gray indicates both SQA and NCV are applicable. Light gray indicates only SQA applies.

A2.2. Question of Interest

In this hypothetical example, an enrichment strategy is undertaken in which an ISCT is performed for a generic edge-to-edge mitral valve repair device, and the results are combined with other information to justify enrollment criteria for a proposed clinical trial in an investigational device exemption (IDE) application.

Question of Interest: *What enrollment criteria should be used for the clinical trial of the generic edge-to-edge mitral valve repair device to prospectively exclude patients who are less likely to respond favorably to the intervention?*

A2.3. Context of Use (COU)

Context of Use: *In this hypothetical enrichment example, an ISCT is performed to provide supporting evidence to establish enrollment criteria for a clinical trial of a generic edge-to-edge mitral valve repair device. Recent evidence shows that mean left atrial pressure measured immediately following edge-to-edge mitral valve repair is an independent predictor of improved patient survival* (20). *To address the question of interest, baseline enrollment criteria are first defined based on prior data. An ISCT is then performed to predict acute post-operative mean left atrial pressure in a virtual patient cohort, and a mapping function converts mean left atrial pressure to predicted mortality (**Figure A7**). The ISCT results inform revisions to the baseline enrollment criteria to prospectively exclude patients who are less likely to respond favorably to the intervention. In this hypothetical example, the ISCT only informs enrollment criteria refinement; it does not influence any other aspects of the clinical trial. In the event that the real human clinical trial successfully demonstrates that the device is safe and effective, the device would be indicated for the same patient population studied in the trial.*

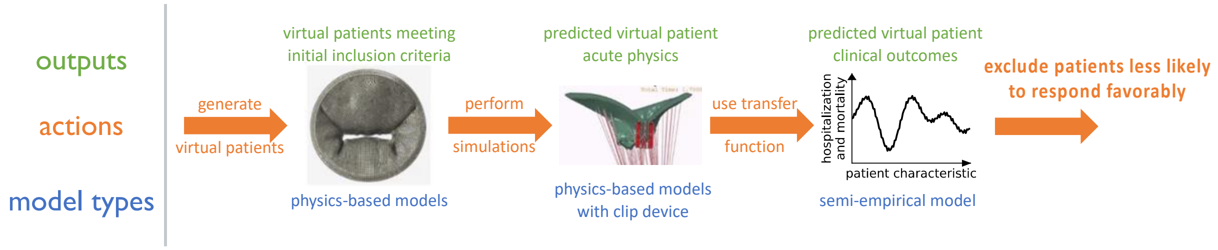

Figure A7: Graphical summary of the context of use for the hypothetical ISCT of an edge-to-edge mitral valve repair device.

A2.4. Model Risk Assessment

A2.4.1. Model Influence

In addition to ISCT evidence, other information will be used to define the trial enrollment criteria, including pre-clinical experiences in animal studies, enrollment criteria for clinical trials performed for other similar devices, and clinical judgement. The overall strategy consists of defining baseline enrollment criteria based on these other sources of information and then using the results of the ISCT to refine the criteria to prospectively exclude patients who are less likely to respond favorably to the intervention. Given this overall strategy, the ISCT evidence has a <u>moderate influence</u> on the decision, but it is <u>weighted less heavily</u> than the other supporting evidence. From the example gradation of

possible model influence scenarios for an ISCT in Table 4 of the main document, this corresponds to a model influence of **Low-Medium**.

Please note that an assessment of **Low-Medium** model influence does not imply that the ISCT evidence is unimportant. Rather, the ISCT evidence may contribute to strengthening the hypothetical investigational device exemption (IDE) submission toward a favorable benefit-to-risk determination.

***Model Influence:* Low-Medium**

A2.4.2. Decision Consequence

For this hypothetical example, the decision tree flowchart shown in Figure 4 of the main document is used to show how the ISCT results inform the selection of enrollment criteria for the proposed real human clinical trial and the potential consequences. As illustrated, potential patient harm could result from an incorrect decision concerning the question of interest, which is: *"What enrollment criteria should be used for the clinical trial of the generic edge-to-edge mitral valve repair device to prospectively exclude patients who are less likely to respond favorably to the intervention?"*

To assess the decision consequence, we consider what patient harm could stem from a bad choice of the enrollment criteria for the proposed clinical trial. In this case, the potential hazardous situation is that of selecting a patient population that will not benefit from the mitral valve repair device, but will potentially suffer procedure- or device-related adverse events that may include (23):

- Single leaflet device attachment
- Device embolization
- Endocarditis requiring surgery
- Mitral valve stenosis
- Myocardial perforation
- Iatrogenic atrial septal defect
- Need for valve replacement rather than repair due in part to procedure- or device-related complications

Following ISO 14971:2019 (24), to evaluate decision consequence we consider the potential *severity* and *probability of occurrence* of harm for such potential adverse events. Using the qualitative levels listed in Table 5 of the main document, the potential *severity* of harm is estimated to be **"Critical"** given that many of the foregoing adverse events are potentially life threatening (e.g., device embolization). To estimate the *probability of occurrence* of harm, we use the device-related complication rate of 3.4% that was reported for a similar marketed device by Stone et al. (17). From Table 6 of the main document, this corresponds to an estimated *probability of occurrence* level of **"Probable."** Combining the *severity* and *probability of occurrence* estimates, using the 5×5 matrix in **Figure A8**, we assess that the overall decision consequence is **Medium-High**.

***Decision Consequence:* Medium-High**

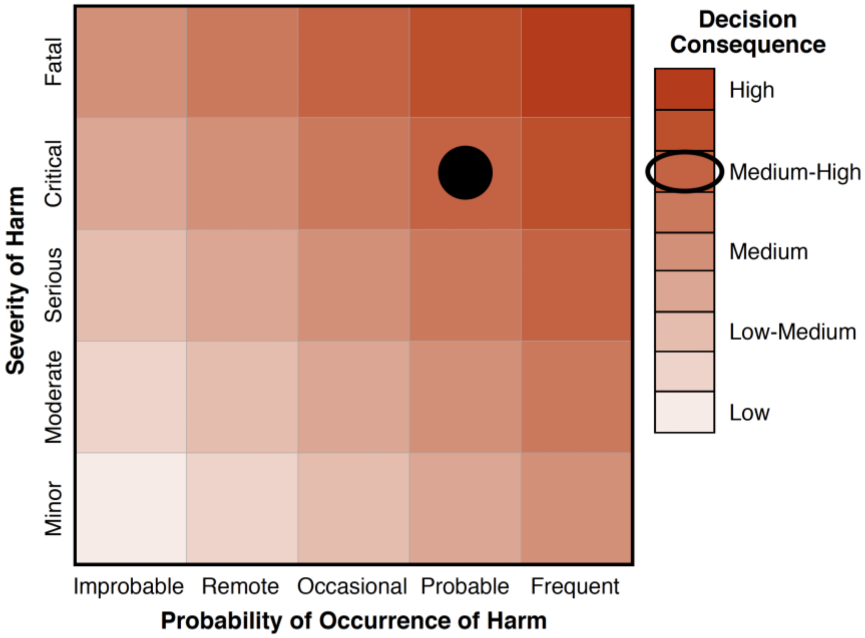

Figure A8: Assessment of decision consequence for the hypothetical ISCT of an edge-to-edge mitral valve repair device.

A2.4.3. Model Risk

To summarize, the model influence and decision consequence for a hypothetical ISCT of a generic edge-to-edge mitral valve repair device were estimated to be:

***Model Influence:* Low-Medium**

***Decision Consequence:* Medium-High**

To assess the model risk, we combine these estimates in **Figure A9**. Here we see that the model risk is estimated to be **Medium.**

***Model Risk:* Medium**

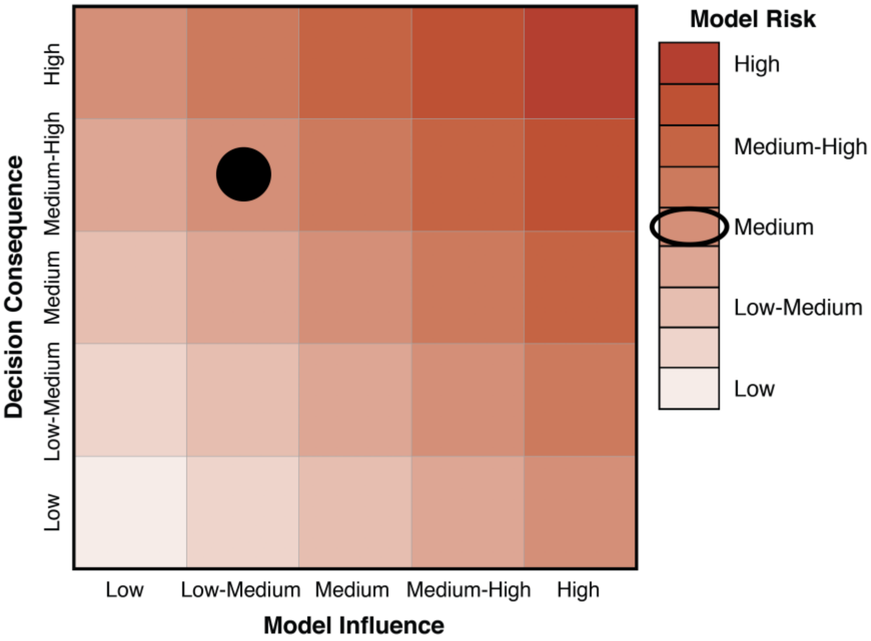

Figure A9: Assessment of model risk for the hypothetical ISCT of an edge-to-edge mitral valve repair device.

A2.5. Credibility Evidence

A2.5.1. Device Model

No example provided. There are several studies in the literature covering different credibility assessment activities for device models (e.g., (25–31)).

A2.5.2. Patient Model

Evidence Category: ② - Model calibration evidence

Summary of Credibility Activity: Patient model parameters are calibrated and the ability of the calibrated model to predict clinical measurements for a single patient with secondary mitral valve regurgitation is assessed.

Calibration approach: The patient model as described in Section A1.2 is used for the calibration simulations, with the exception that the leaflet geometries are generated based on patient-specific cardiac computed tomography data. In brief, computed tomography images at diastole are manually segmented using iTK-Snap and then processed in 3DEXPERIENCE as follows: triangulated surface meshes corresponding to the ventricular side of the mitral valve leaflets are extracted, three-dimensional leaflet volumes are created by extruding the surface mesh uniformly in the normal (atrial) direction, and finite element volume meshes are generated. The locations of the nodes on the leaflet annulus and those that correspond to the attachment points of the chordae to the papillary muscles are also extracted from the clinical images at end systole and end diastole, and time-varying displacement boundary conditions are specified to control their positions during simulated cardiac cycles.

To perform the calibration, a preliminary sensitivity study of the lumped parameter hemodynamic model is first used to identify the ten input parameters (out of 92 in total) with the greatest influence on

clinical quantities of interest. The identified influential parameters are then calibrated in the patient model co-simulation using an automated multi-objective optimization routine to minimize the difference between the simulation predictions and clinical measurements listed in **Table A4**. During this step, the initial chordae configuration is estimated based on patient images and anatomical studies from the literature. Fixed ranges are also specified for the ten calibrated parameters to limit the search space to physiological ranges based on reference values from literature. Finally, after the automated calibration procedure, ad-hoc manual tuning of the chordae configuration and leaflet geometries is performed to minimize the error between the predicted and segmented shapes of the leaflets during systole.

Data (clinical): Comparator data for model calibration includes pre-operative transesophageal echocardiography, transthoracic echocardiography, computed tomography (191 frames with voxel size 0.488 x 0.488 x 1 mm for each of 10 cardiac time points), and catheterization measurements. All data were obtained under Institutional Review Board (IRB) approval from Boston Children's Hospital and Washington University following patient consent.

Final calibration assessment: After calibrating the model, the final patient model response and the clinical calibration data are compared quantitatively and qualitatively. Quantitative outputs that are compared are either direct outputs of the patient model or are calculated based on one or more direct outputs. Two categorical outputs are also compared: (i) mitral regurgitation grade, estimated based on the predicted regurgitant volume (9), and (ii) stenosis, evaluated based on mitral valve area at diastole (32). For numerical quantities of interest, a percent difference is calculated using the clinical data as the referent.

Summary of Results: Following calibration, predicted leaflet shapes in diastole and systole qualitatively match those observed from clinical transesophageal echocardiography imaging (**Figure A10**). Predicted and observed categorical measurements agree (**Table A4**), and percent differences in predicted quantities of interest range from -19% to +16% (**Table A4**).

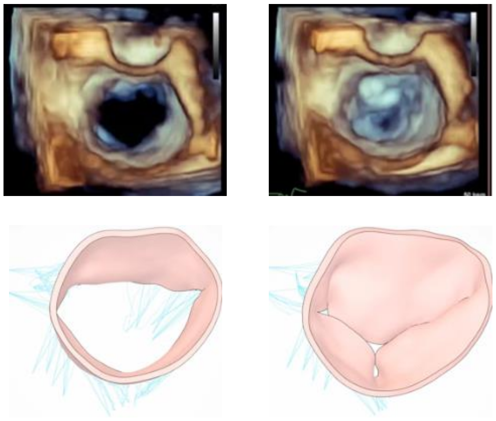

Figure A10*:* Qualitative comparison of three-dimensional transesophageal echocardiography (top) and patient-specific computational simulation (bottom) showing the mitral valve in diastole (left) and systole (right). The diastolic state from the clinical imaging data and the model demonstrates adequate leaflet opening with unobstructed inflow. In systole, echocardiography demonstrates a central to posterior coaptation defect as a source of regurgitation. The model predicts a corresponding central to posterior coaptation defect consistent with a common pseudocleft defect in the posterior leaflet as an etiology of the regurgitation in this patient.

Table A4*:* Quantitative comparison of patient data measured clinically and that predicted computationally after final parameter calibration. LV: left ventricle; LA: left atrium; EF: ejection fraction; ED: end-diastolic; ES: end-systolic; TTE: transthoracic echocardiography; CT: computed tomography

Attribute	***Clinical Data Source***	***Clinical Measurement***	***Patient Model Prediction***	***Percent Difference***
LV EF	TTE	0.41	0.39	-5%
LV ED vol (ml)	CT	290	278	-4%
LV ES vol (ml)	CT	201	169	-16%
LA ED vol (ml)	CT	184	214	+16%
LA ES vol (ml)	CT	196	158	-19%
LA mean pressure (mmHg)	Catheter	24	23.3	-3%
LA V wave pressure (mmHg)	Catheter	44	45	-3%
Mitral Regurgitation Grade	Clinician	Severe	Severe	N/A
Stenosis	Clinician	None	None	N/A

Limitations and Gaps:

- The calibration activity relies on data for a single patient with secondary mitral valve regurgitation. Some model parameters may be universally applicable such that calibration on a limited patient data set can be justified. However, in practice, robustly establishing input parameters via calibration will likely involve considering multiple patients spanning the patient population, or possibly several separate calibration activities to establish appropriate input parameters for specific patient sub-populations.
- Following calibration, final percent difference magnitudes for predicted quantities of interest remain as large as approximately 20%. This indicates not all parameters could be simultaneously calibrated to closely match clinical measurements. Potential reasons for the large percent differences include limitations in the ability of the model to resolve all relevant physics (i.e., model form error), incorrect estimation of physiological limits for the calibrated parameters, limitations of the calibration procedure itself to identify a global optimum parameter set within the multivariate search space, or inappropriate values used for the fixed (less influential) parameters of the model.
- The calibration activity does not consider uncertainty in the calibration data. Clinical measurements considered herein rely on imaging data with limited spatial resolution and potential errors in temporal gating. Variability in measurements across cardiac cycles is also not estimated or considered. Thus, errors in clinical calibration measurements may bias the calibrated parameters and reduce the accuracy of the model for predictions away from calibration conditions.
- Even in ideal scenarios using extremely precise calibration measurements and robust multivariate nonlinear optimization approaches, calibration does not guarantee the predictive accuracy of a computational model away from calibration conditions. Independent validation activities covering a range of patients representative of the target population would strengthen the overall model credibility evidence and help to confirm calibrated parameters are appropriate.

A2.5.3. Coupled Device-Patient Model

Evidence Category: ④ - In vivo validation

Summary of Credibility Activity: Coupled device-patient model predictions are compared with post-operative clinical measurements of a single patient following edge-to-edge mitral valve repair. The patient is the same as that studied in the Section A2.5.2 example. The generic device model is modified by adjusting the width of the arms to reflect the specific device used to treat this patient.

Validation simulations: The acute post-operative (clipped) state of the patient model is simulated as described in Section A1.3 using the same patient valve geometry described in Section A2.5.2.

Data (clinical): Comparator data for this validation exercise include acute post-operative transesophageal echocardiography, transthoracic echocardiography images, and catheterization measurements following edge-to-edge mitral valve repair using a single device. All data were obtained under IRB approval from Boston Children's Hospital and Washington University following patient consent.

Method of comparison: Coupled device-patient model predictions and post-operative clinical data are compared quantitatively and qualitatively (similar to the approach presented in Section A2.5.3). Quantitative outputs that are compared are either direct outputs of the coupled device-patient model or are calculated based on one or more direct outputs. Two categorical outputs are also compared: (i) mitral regurgitation grade, estimated based on the predicted regurgitant volume (9), and (ii) stenosis, evaluated based on mitral valve area at diastole (32). For numerical quantities of interest, a percent difference is calculated using the clinical data as the referent.

Summary of Results: Post-procedural simulation predictions of leaflet shapes in diastole and systole qualitatively match those observed in clinical transesophageal echocardiography imaging (**Figure A11**). Simulations during diastole predict a dual orifice shape that is also observed in the clinical data (**Figure A11**). Percent differences in predicted quantities of interest range from -9% to +40% (**Table A5**). Categorical predictions agree for stenosis (none) but differ by one gradation level for mitral regurgitation grade ('trace' regurgitation observed clinically versus 'mild' regurgitation predicted by simulation; **Table A5**).

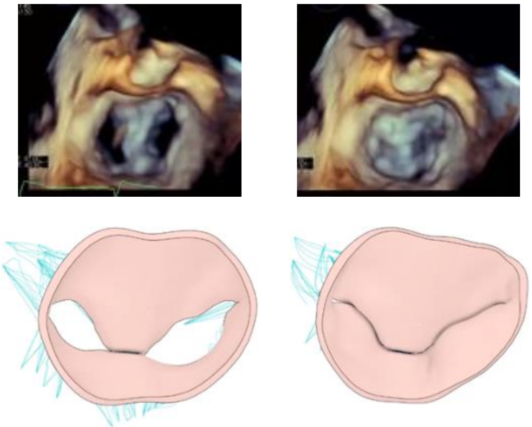

Figure A11: Qualitative comparison of the post-procedural mitral valve geometry in the open (left) and closed (right) configurations from clinical transesophageal echocardiography (top) and computational simulation (bottom). Note that the post-operative dual orifice appearance of the open mitral valve predicted by the computational model under systolic conditions approximately matches the appearance of the valve in the clinical imaging data.

Table A5*:* Quantitative comparison of patient data measured clinically and that predicted computationally. LV: left ventricle; LA: left atrium; EF: ejection fraction; TTE: transthoracic echocardiography; Cath: catheter; TEE: transesophageal echocardiography

Attribute	*Clinical Data Source*	*Clinical Measurement*	*Patient Model Prediction*	*Percent Difference*
LV EF	TTE	0.31	0.35	+13%
LA mean pressure (mmHg)	Catheter	22	19.3	-12%
LA V wave pressure (mmHg)	Catheter	34	31.1	-9%
Mitral valve diastolic pressure gradient (mmHg)	TEE	3	4.2	+40%
Mitral Regurgitation Grade	Clinician	Trace	Mild	N/A
Stenosis	Clinician	None	None	N/A

Limitations and Gaps:

- The validation activity relies on post-operative comparator data from a single patient that would likely be inadequate to support model credibility in practice. For a real scenario, the adequacy of the validation evidence could be systematically assessed by adapting ASME V&V 40-2018 validation credibility factors for patient-specific modeling as proposed in Galappaththige et al. (5). For example, ASME V&V 40-2018 credibility sub-factors '*quantity of test samples*' and '*range of characteristics of test samples*' could be revised to '*number of validation subjects*' and '*range and characteristics of validation subjects,*' respectively (see Table 5 in (5)). Using these revised credibility factors, goals for the number and range of patients to include in the validation activity could be established based on the model risk, and later reviewed as part of prospective and post-study adequacy assessments (1).
- Using the previously calibrated input parameters for the patient model (Section A2.5.2), the coupled patient-device model predictions agree with clinical measurements within approximately 10% for all but one quantity of interest, mitral valve diastolic pressure gradient. For the latter quantity, model predictions are 40% higher than the comparator measurements. For a real ISCT application, credibility factors from ASME V&V 40-2018 could again be useful to systematically assess the validation evidence (5). For example, goals for sub-factor '*agreement of output comparison*' could be prospectively established and later assessed following the validation exercise. If the goal is not met – for example, if 40% error in mitral valve diastolic pressure gradient is not acceptable – then the model may need to be revised, the validation measurement accuracy improved, or the overall model context of use modified to account for limitations in model accuracy (1,2).
- As in Section A2.5.2, no uncertainty quantification is performed for input parameters, model predictions, or clinical measurements. All inputs are fixed, and the validation exercise relies on a single deterministic simulation. Credibility factors from ASME V&V 40-2018 could once again be adapted (as in (5)) to establish goals for and assess the adequacy of uncertainty quantification activities performed to support the coupled device-patient model validation.

A2.5.4. Virtual Patient Cohort Model

Evidence Category: ⑦ - Model plausibility

Summary of Credibility Activity: The plausibility of the virtual patient cohort input parameters is supported by literature data characterizing mitral valve anatomy. In brief, baseline locations of each virtual patient's papillary muscles and their position relative to one another (interpapillary distance) and the annular plane (height) are established using information from Veronesi et al. (33). The interpapillary distance and height are also varied as boundary conditions guided by data from Veronesi et al. (33). The initial size and shape of each virtual patient's mitral valve annulus are defined using geometric parameters from several previous studies (34–36), and the radial displacements of nodes on the annulus are specified as boundary conditions guided by the literature (34–36).

Limitations and Gaps:

- The identified plausibility evidence only qualitatively confirms that input parameters are reasonable. The evidence does not validate that the virtual patients are representative of real patients.
- Uncertainties in the referenced literature measurements are not considered.
- Each literature study referenced involves a different patient cohort. Combining parameter measurements from multiple studies could lead to the generation of unrealistic virtual patients, especially when parameter correlations are not considered.

Evidence Category: N/A - Expert Judgement

As noted in the FDA CM&S Credibility Guidance (1), the provided categorization of evidence *"is not intended to be exhaustive. In some cases, there may be a need to define new categories if the credibility evidence does not fit into any of the [existing] categories."* Thus, in addition to Category ⑦ evidence, we also evaluated the realism of the virtual patient cohort model output based on the expert clinical judgment of an experienced transcatheter edge-to-edge repair interventionalist.

Summary of Credibility Activity: The realism of the virtual patient cohort leaflet anatomy, behavior, and mitral valve regurgitation grade are supported by expert clinical judgment. In brief, virtual patients are generated (as described in Section A1.4) and simulations are performed (as described in Section A1.2) to predict regurgitant volume (**Table A6** and **Figure A12**). The mitral valve regurgitation grade level is then estimated for each member of the initial virtual patient cohort from the predicted regurgitant volume based on values from the clinical literature (9). Mitral valve morphology is examined and each virtual patient is categorized into one of four major classes (**Table A7**) from which representative samples are extracted (**Figure A13**). An experienced transcatheter edge-to-edge repair interventionalist is then consulted to review the sampled virtual patients to confirm that the leaflet configurations, coaptation behavior, and predicted mitral regurgitation grades are clinically realistic.

Table A6: Statistics for the initial virtual patient cohort. MR: mitral regurgitation; RVOL: regurgitant volume.

MR Grade Attribute	Mean	Std Dev	Median	Min	Max
RVOL (ml)	50.5	24.1	51.5	9.3	121.9

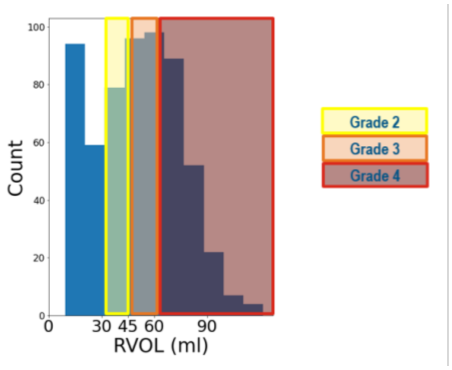

Figure A12: Distributions of regurgitant volume (RVOL) for the initial virtual patient cohort.

Table A7: Classifications for the virtual patients in the initial virtual patient cohort. AL: anterior leaflet; PL: posterior leaflet

Type	Description
1	Restrictive or shortened AL
2	Restrictive or shortened PL
3	AL and PL free edges at same height along coaptation line with little or no coaptation (< 2mm)
4	AL and PL free edges at same height along coaptation line with substantive coaptation (> 2mm)

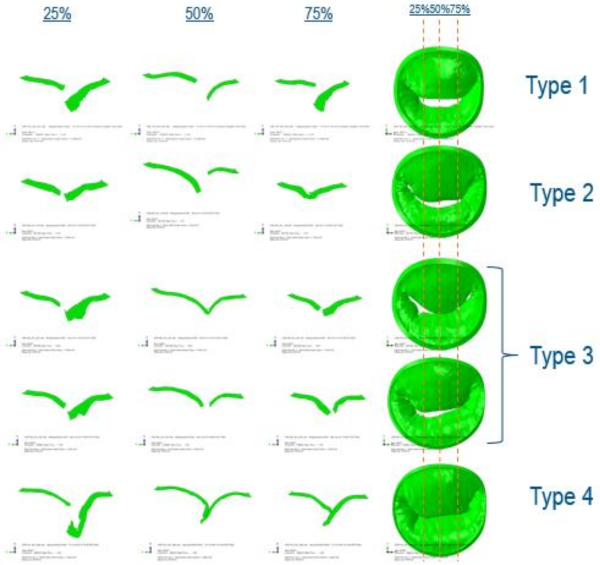

Figure A13: Representative samples for each mitral valve classification type. Such representative samples are reviewed by a real clinician to assess whether the valve geometries, predicted leaflet behaviors, and predicted regurgitation grades are clinically realistic.

Limitations and Gaps:

- The presented evidence only qualitatively supports that the generated virtual patient morphologies and their associated coaptation behavior and regurgitant volumes are clinically realistic representations of pathological patients. The evidence does not validate the virtual patients. Furthermore, validating virtual cohorts composed of synthetic patients remains a scientific challenge, as individual patients cannot be directly validated given "*there is not a corresponding real patient to compare against*" (15). Instead, cohorts could be "*validated in a statistical manner by comparing cohort-level statistics against population-level statistics*" (15), which would correspond to Category ⑤ credibility evidence (see Table 1 of the main document).
- Only a single clinician was consulted to review the realism of the generated patients. The evidence could be strengthened by review from multiple clinicians with experience in transcatheter edge-to-edge repair.

A2.5.5. Clinician Model

Evidence Category: ⑦ - Model plausibility

Summary of Credibility Activity: The clinician model described in Section A1.5 was developed through consultation with an interventional cardiologist and following published clinical consensus guidelines and recommendations for transcatheter edge-to-edge repair (9,32,37,38). The model utilizes quantitative criteria to guide clipping and to assess patient acute outcomes. Our decision tree for clipping uses thresholds for transmitral pressure gradient and the reduction in mitral regurgitation grade level to determine the treatment course. Transmitral pressure gradient is recognized as a critical clinical quantity for stenosis, and a threshold ceiling is used as a safeguard against inducing stenosis upon clipping (32). Successful treatment has been recognized as one that achieves a reduction of mitral regurgitation to a grade level ≤ 2 and does not induce stenosis (32,37). The clinician model incorporates both considerations in the treatment decision tree. Additionally, when simulating virtual patients, an interventional cardiologist was consulted in the initial placement of the device in a subset of the patients to ensure that the intervention was clinically realistic.

Limitations and Gaps:

- The presented evidence only qualitatively supports the plausibility of the clinician model. The evidence does not validate the clinician model.
- Some aspects of the clinical intervention are difficult to translate directly into physics-based input quantities and boundary conditions. Although a decision tree was developed based on established clinical best practices, the model may misrepresent a real intervention for some clinical scenarios (see also Limitations and Gaps in Section A1.5).
- Only a single clinician was consulted to assess the initial device placement. Consultation with several clinicians could help ensure the model adequately accounts for variability in surgical approaches.
- No clinician input was provided on final simulated interventions. Additional review by expert clinicians with relevant experience would strengthen the credibility of the clinician model.

A2.5.6. Clinical Outcome Mapping Model

Evidence Category: ⑦ - Model plausibility

Summary of Credibility Activity: The clinical outcome mapping model is based on recent observations by El Shaer et al. (20). In this study, the authors separated 463 patients who underwent transcatheter edge-to-edge repair at the Mayo Clinic from 2014-2021 according to mean post-procedural left atrial pressure (LAP). The high LAP group had LAP > 22 mmHg and the low group LAP ≤ 22 mmHg. The investigators examined all-cause mortality over the follow-up period and compared survival rates using Kaplan Meier and Cox proportional hazard analyses. The study concluded patients in the lower post-operative LAP group had significantly higher survival probability (hazard ratio=1.71 [95% CI, 1.10–2.70], p=0.02; see Figure 1 in (20) and accompanying text). These data from the El Shaer et al. (20) study provide model plausibility evidence for the correlation between acute values of LAP and patient survival.

Limitations and Gaps:

- The limitations and gaps of the provided plausibility evidence are similar to those mentioned previously in the clinical outcome mapping model description (see Section A1.6). In brief, although the referenced study establishes a correlation between a clinically measurable physics-based quantity (left atrial pressure) and a clinical outcome (patient mortality), the observation of a correlation in a single study does not validate the mapping for all patient populations. Clinical outcome mapping validation would ideally follow using data from a second, independent clinical study. The general strength and uncertainty of the correlation used in the mapping model are also difficult to infer from a single literature study.

A2.5.7. Full ISCT

No example provided.

References

1. FDA. Assessing the Credibility of Computational Modeling and Simulation in Medical Device Submissions: Guidance for Industry and Food and Drug Administration Staff. (2023). https://www.fda.gov/media/154985/download

2. ASME V&V 40-2018. Assessing Credibility of Computational Modeling Through Verification and Validation: Application to Medical Devices. New York, NY: American Society of Mechanical Engineers. (2018).

3. Pathmanathan P, Aycock KI, Badal A, Bighamian R, Bodner J, Craven BA, Niederer S. Credibility assessment of in silico clinical trials for medical devices. *PLOS Comput Biol* (2024) (in press): doi: 10.1371/journal.pcbi.1012289

4. Bischoff JE, Dharia MA, Favre P. A risk and credibility framework for in silico clinical trials of medical devices. *Comput Methods Programs Biomed* (2023) 242:107813. doi: 10.1016/j.cmpb.2023.107813

5. Galappaththige S, Gray RA, Mendonca Costa C, Niederer S, Pathmanathan P. Credibility assessment of patient-specific computational modeling using patient-specific cardiac modeling as an exemplar. *PLOS Comput Biol* (2022) 18:e1010541. doi: 10.1371/journal.pcbi.1010541

6. Pibarot P, Delgado V, Bax JJ. MITRA-FR vs. COAPT: lessons from two trials with diametrically opposed results. *Eur Heart J Cardiovasc Imaging* (2019) 20:620–624. doi: 10.1093/ehjci/jez073

7. Dassault Systèmes Simulia Corporation. Abaqus Analysis Guide. Johnston, RI. (2022).

8. Dassault Systèmes Simulia Corporation. Dymola User Manual. Johnston, RI. (2022).

9. Zoghbi WA, Adams D, Bonow RO, Enriquez-Sarano M, Foster E, Grayburn PA, Hahn RT, Han Y, Hung J, Lang RM, et al. Recommendations for noninvasive evaluation of native valvular regurgitation: A report from the American Society of Echocardiography developed in collaboration with the Society for Cardiovascular Magnetic Resonance. *J Am Soc Echocardiogr* (2017) 30:303–371. doi: 10.1016/j.echo.2017.01.007

10. Toma M, Einstein DR, Bloodworth CH, Cochran RP, Yoganathan AP, Kunzelman KS. Fluid–structure interaction and structural analyses using a comprehensive mitral valve model with 3D chordal structure. *Int J Numer Methods Biomed Eng* (2017) 33:e2815. doi: 10.1002/cnm.2815

11. Toma M, Einstein DR, Kohli K, Caroll SL, Bloodworth CH, Cochran RP, Kunzelman KS, Yoganathan AP. Effect of edge-to-edge mitral valve repair on chordal strain: fluid-structure interaction simulations. *MDPI Biology* (2020) 9:173. doi: 10.3390/biology9070173

12. Sacks MS, Drach A, Lee C-H, Khalighi AH, Rego BV, Zhang W, Ayoub S, Yoganathan AP, Gorman RC, Gorman JH. On the simulation of mitral valve function in health, disease, and treatment. *J Biomech Eng* (2019) 141:070804. doi: 10.1115/1.4043552

13. Toma M, Einstein D, Bloodworth C, Kohli K, Cochran R, Kunzelman K, Yoganathan A. Fluid-structure interaction analysis of subject-specific mitral valve regurgitation treatment with an intra-valvular spacer. *MDPI Prosthesis* (2020) 2:65–75. doi: 10.3390/prosthesis2020007

14. Faris O, Shuren J. An FDA viewpoint on unique considerations for medical-device clinical trials. *N Engl J Med* (2017) 376:1350–1357. doi: 10.1056/NEJMra1512592

15. Niederer SA, Aboelkassem Y, Cantwell CD, Corrado C, Coveney S, Cherry EM, Delhaas T, Fenton FH, Panfilov AV, Pathmanathan P, et al. Creation and application of virtual patient cohorts of heart models. *Phil Trans R Soc A* (2020) 378:20190558. doi: 10.1098/rsta.2019.0558

16. Neuss M, Schau T, Isotani A, Pilz M, Schöpp M, Butter C. Elevated mitral valve pressure gradient after MitraClip implantation deteriorates long-term outcome in patients with severe mitral regurgitation and severe heart failure. *JACC Cardiovas Interv* (2017) 10:931–939. doi: 10.1016/j.jcin.2016.12.280

17. Stone GW, Lindenfeld J, Abraham WT, Kar S, Lim DS, Mishell JM, Whisenant B, Grayburn PA, Rinaldi M, Kapadia SR, et al. Transcatheter mitral-valve repair in patients with heart failure. *N Engl J Med* (2018) 379:2307–2318. doi: 10.1056/NEJMoa1806640

18. Corrigan FE, Chen JH, Maini A, Lisko JC, Alvarez L, Kamioka N, Reginauld S, Gleason PT, Condado JF, Wei JW, et al. Pulmonary venous waveforms predict rehospitalization and mortality after percutaneous mitral valve repair. *JACC Cardiovasc Imaging* (2019) 12:1905–1913. doi: 10.1016/j.jcmg.2018.07.014

19. Grayburn PA, Sannino A, Packer M. Proportionate and disproportionate functional mitral regurgitation. *JACC Cardiovasc Imaging* (2019) 12:353–362. doi: 10.1016/j.jcmg.2018.11.006

20. El Shaer A, Thaden J, Eleid M, Simard T, Guerrero M, Rihal CS, Alkhouli M. Hemodynamic success is an independent predictor of mid-term survival after transcatheter edge-to-edge mitral valve repair. *Circ Cardiovasc Interv* (2022) 15:e011542. doi: 10.1161/CIRCINTERVENTIONS.121.011542

21. Pathmanathan P, Gray RA. Validation and trustworthiness of multiscale models of cardiac electrophysiology. *Front Physiol* (2018) 9:106. doi: 10.3389/fphys.2018.00106

22. Hills RG, Pilch M, Dowding KJ, Red-Horse J, Paez TL, Babuška I, Tempone R. Validation challenge workshop. *Comput Methods in Appl Mech Eng* (2008) 197:2375–2380. doi: 10.1016/j.cma.2007.10.016

23. ClinicalTrials.gov. *Identifier NCT01626079, Cardiovascular Outcomes Assessment of the MitraClip Percutaneous Therapy for Heart Failure Patients With Functional Mitral Regurgitation (The COAPT Trial) and COAPT CAS (COAPT).* Bethesda, MD: National Library of Medicine. (2012). https://clinicaltrials.gov/study/NCT01626079

24. ISO 14971:2019. Medical devices — Application of risk management to medical devices. Geneva, Switzerland: International Standards Organization. (2019).

25. Aycock KI, Rebelo N, Craven BA. Method of manufactured solutions code verification of elastostatic solid mechanics problems in a commercial finite element solver. *Comput Struct* (2020) 229:106175. doi: 10.1016/j.compstruc.2019.106175

26. Aycock KI, Rebelo N, Craven BA. Method of exact solutions code verification of a superelastic constitutive model in a commercial finite element solver. *Adv Eng Softw* (2024) 191:103609. doi: 10.1016/j.advengsoft.2024.103609

27. Guler I, Aycock KI, Rebelo N. Two calculation verification metrics used in the medical device industry: Revisiting the limitations of fractional change. *J Verif Valid Uncertainty Quantif* (2022) 7:031004. doi: 10.1115/1.4055506

28. Ponnaluri SV, Hariharan P, Herbertson LH, Manning KB, Malinauskas RA, Craven BA. Results of the interlaboratory computational fluid dynamics study of the FDA benchmark blood pump. *Ann Biomed Eng* (2023) 51:253–269. doi: 10.1007/s10439-022-03105-w

29. Wanki G, Ekwaro-Osire S, Dias JP, Cunha A. Uncertainty quantification with sparsely characterized parameters: An example applied to femoral stem mechanics. *J Verif Valid Uncertainty Quantif* (2020) 5:031005. doi: 10.1115/1.4048749

30. Santiago A, Butakoff C, Eguzkitza B, Gray RA, May-Newman K, Pathmanathan P, Vu V, Vázquez M. Design and execution of a verification, validation, and uncertainty quantification plan for a numerical model of left ventricular flow after LVAD implantation. *PLOS Comput Biol* (2022) 18:e1010141. doi: 10.1371/journal.pcbi.1010141

31. Nagaraja S, Loughran G, Baumann AP, Kartikeya K, Horner M. Establishing finite element model credibility of a pedicle screw system under compression-bending: An end-to-end example of the ASME V&V 40 standard. *Methods* (2024) 225:74–88. doi: 10.1016/j.ymeth.2024.03.003

32. Baumgartner H, Hung J, Bermejo J, Chambers JB, Evangelista A, Griffin BP, Iung B, Otto CM, Pellikka PA, Quinones M. Echocardiographic assessment of valve stenosis: EAE/ASE recommendations for clinical practice. *Eur J Echocardiogr* (2009) 10:1–25. doi: 10.1093/ejechocard/jen303

33. Veronesi F, Corsi C, Sugeng L, Caiani EG, Weinert L, Mor-Avi V, Cerutti S, Lamberti C, Lang RM. Quantification of mitral apparatus dynamics in functional and ischemic mitral regurgitation using real-time 3-dimensional echocardiography. *J Am Soc Echocardiogr* (2008) 21:347–354. doi: 10.1016/j.echo.2007.06.017

34. Topilsky Y, Vaturi O, Watanabe N, Bichara V, Nkomo VT, Michelena H, Le Tourneau T, Mankad SV, Park S, Capps MA, et al. Real-time 3-dimensional dynamics of functional mitral regurgitation: A prospective quantitative and mechanistic study. *J Am Heart Assoc* (2013) 2:e000039. doi: 10.1161/JAHA.113.000039

35. Morbach C, Bellavia D, Störk S, Sugeng L. Systolic characteristics and dynamic changes of the mitral valve in different grades of ischemic mitral regurgitation – insights from 3D transesophageal echocardiography. *BMC Cardiovasc Disord* (2018) 18:93. doi: 10.1186/s12872-018-0819-z

36. van Wijngaarden SE, Kamperidis V, Regeer MV, Palmen M, Schalij MJ, Klautz RJ, Bax JJ, Ajmone Marsan N, Delgado V. Three-dimensional assessment of mitral valve annulus dynamics and impact on quantification of mitral regurgitation. *Eur Heart J Cardiovasc Imaging* (2018) 19:176–184. doi: 10.1093/ehjci/jex001

37. Zoghbi WA, Asch FM, Bruce C, Gillam LD, Grayburn PA, Hahn RT, Inglessis I, Islam AM, Lerakis S, Little SH, et al. Guidelines for the evaluation of valvular regurgitation after percutaneous valve repair or replacement: A report from the American Society of Echocardiography developed in collaboration with the Society for Cardiovascular Angiography and Interventions, Japanese Society of Echocardiography, and Society for Cardiovascular Magnetic Resonance. *J Am Soc Echocardiogr* (2019) 32:431–475. doi: 10.1016/j.echo.2019.01.003

38. Dujardin KS, Enriquez-Sarano M, Bailey KR, Nishimura RA, Seward JB, Tajik AJ. Grading of mitral regurgitation by quantitative Doppler echocardiography: Calibration by left ventricular angiography in routine clinical practice. *Circ* (1997) 96:3409–3415. doi: 10.1161/01.CIR.96.10.3409
